# Regulation of germination by targeted mutagenesis of grain dormancy genes in barley

**DOI:** 10.1111/pbi.13692

**Published:** 2021-09-08

**Authors:** Hiroshi Hisano, Robert E. Hoffie, Fumitaka Abe, Hiromi Munemori, Takakazu Matsuura, Masaki Endo, Masafumi Mikami, Shingo Nakamura, Jochen Kumlehn, Kazuhiro Sato

**Affiliations:** ^1^ Institute of Plant Science and Resources Okayama University Kurashiki Japan; ^2^ Leibniz Institute of Plant Genetics and Crop Plant Research (IPK) Gatersleben Stadt Seeland Germany; ^3^ Institute of Crop Science NARO Tsukuba Japan; ^4^ Institute of Agrobiological Sciences NARO Tsukuba Japan

**Keywords:** *Hordeum vulgare*, seed dormancy, targeted genome modification, CRISPR, Cas9 nuclease, pre‐harvest sprouting

## Abstract

High humidity during harvest season often causes pre‐harvest sprouting in barley (*Hordeum vulgare*). Prolonged grain dormancy prevents pre‐harvest sprouting; however, extended dormancy can interfere with malt production and uniform germination upon sowing. In this study, we used Cas9‐induced targeted mutagenesis to create single and double mutants in *QTL FOR SEED DORMANCY 1* (*Qsd1*) and *Qsd2* in the same genetic background. We performed germination assays in independent *qsd1* and *qsd2* single mutants, as well as in two double mutants, which revealed a strong repression of germination in the mutants. These results demonstrated that normal early grain germination requires both *Qsd1* and *Qsd2* function. However, germination of *qsd1* was promoted by treatment with 3% hydrogen peroxide, supporting the notion that the mutants exhibit delayed germination. Likewise, exposure to cold temperatures largely alleviated the block of germination in the single and double mutants. Notably, *qsd1* mutants partially suppress the long dormancy phenotype of *qsd2*, while *qsd2* mutant grains failed to germinate in the light, but not in the dark. Consistent with the delay in germination, abscisic acid accumulated in all mutants relative to the wild type, but abscisic acid levels cannot maintain long‐term dormancy and only delay germination. Elucidation of mutant allele interactions, such as those shown in this study, are important for fine‐tuning traits that will lead to the design of grain dormancy through combinations of mutant alleles. Thus, these mutants will provide the necessary germplasm to study grain dormancy and germination in barley.

## Introduction

Grains exhibit dormancy to prevent sprouting under adverse conditions and environments and only germinate when favourable conditions return. Grain dormancy in crops has been greatly shortened during domestication to allow repeated sowing and harvesting over a single growing season (Gubler *et al*., 2005). This brevity of dormancy can cause pre‐harvest sprouting in some temperate areas where the crop harvesting season coincides with high humidity. Pre‐harvest sprouting negatively affects agriculture and the economy by lowering grain yield and quality in cereal crops such as rice (*Oryza sativa*), maize (*Zea mays*), wheat (*Triticum aestivum*), barley (*Hordeum vulgare*), and sorghum (*Sorghum bicolor*). Barley has been domesticated for human consumption and is a widely cultivated cereal crop (Sato, 2020). Amongst others, barley is used as raw material for malting, which requires synchronized and rapid germination, but cultivated barley is more prone to pre‐harvest sprouting than its wild form (Li *et al*., 2004). The balance between dormancy and germinability is one of the main goals driving barley breeding programmes.

Grain dormancy is strongly affected by environmental conditions, for example temperature, humidity, light, and nutritional status during grain maturation and germination. For example, during grain maturation, high and low temperatures result in shorter and longer dormancy, respectively, in barley and wheat (Reddy *et al*., 1985; Schuurink *et al*., 1992). However, low temperatures prevent germination in rice cultivated in the summer season, while high temperatures repress germination in barley and wheat cultivated in the winter–spring season.

Grain dormancy is a quantitative trait regulated by many genes and metabolites. Among these factors, the endogenous levels of and sensitivity to two antagonistic phytohormones, abscisic acid (ABA) and gibberellic acid (GA), play critical roles in controlling dormancy and germination (Graeber *et al*., 2012). ABA is one of the most important regulators of grain dormancy and development, and it suppresses germination (McCarty, 1995). ABA levels increase and peak during grain maturation, leading to longer dormancy. Conversely, dormancy is gradually released after reaching full physiological maturity by the induction of reactive oxygen species (ROS) caused by higher levels of GA. Thus, mutants involved in grain dormancy have been used to study ABA and GA synthesis, metabolism, and sensitivity genes, which include those encoding the entire downstream signalling pathway originating from hormone receptors. However, due to the pleiotropic effects of hormones, these mutant alleles are difficult to use in crop breeding. Therefore, the use of genes for natural variation of grain dormancy and their precise control are required for future improvement.

The identification of natural polymorphisms may help improve pre‐harvest sprouting tolerance (Nakamura, 2018). Since the first report of a quantitative trait locus (QTL) for grain dormancy in barley (Ullrich *et al*., 1993), multiple additional QTL have been described that map across all chromosomes using several barley accessions as genetic test material (Hickey *et al*., 2012; Hori *et al*., 2007; Li *et al*., 2003; Nakamura *et al*., 2017; Sato *et al*., 2009). Of those, two major dormancy‐related loci were identified and named *SEED DORMANCY 1* (*SD1*) and *SD2* (Han *et al*., 1996). The causal gene for *SD1* was identified by a map‐based cloning approach using barley cv. ‘Haruna Nijo’ and wild barley ‘OUH602’ and was renamed *QTL FOR SEED DORMANCY 1* (*Qsd1*). *Qsd1* is predicted to encode an alanine aminotransferase (Sato *et al*., 2016). An analysis of the association between genotype and phenotype across barley germplasm showed that the single nucleotide polymorphism G642C in exon 9 of *Qsd1*, causing the L214F amino acid change, is associated with grain dormancy; this polymorphism is thought to have originated in West Asia after domestication and helped usher in the transition from long dormancy in wild barley to short dormancy in cultivated barley (Sato *et al*., 2016).

The identity of *Qsd1* was independently confirmed with transgenic techniques. First, grains from transgenic lines expressing an RNA interference (RNAi) construct that lowered *Qsd1* mRNA in the cultivar ‘Golden Promise’, which carries a wild‐type functional *Qsd1* allele, were suppressed in their germination (Sato *et al*., 2016). In addition, a backcross line harbouring a chromosome fragment derived from ‘OUH602’ and encompassing the *qsd1* long dormancy allele in ‘Golden Promise’ background was generated; then the *Qsd1* gene from cultivar ‘Haruna Nijo’ was transformed into this backcross line, resulting complementation of the short dormancy phenotype (Sato *et al*., 2016).

*SD2* (renamed *Qsd2*) was also cloned by map‐based cloning using barley cvs. ‘Azumamugi’ and ‘Kanto Nakate Gold’ and was shown to encode MITOGEN‐ACTIVATED PROTEIN KINASE KINASE 3 (MAPKK3) (Nakamura *et al*., 2016). In the case of *Qsd2*, the N260T amino acid change in the dormant ‘Azumamugi’ allele was shown to be causal. Additional *qsd2* mutants generated by ethyl methanesulfonate (EMS) mutagenesis in cv. ‘Barke’ were also described: premature termination of *Qsd2* translation was associated with reduced germination. For *Qsd2*, there have been no functional confirmation experiments using genetic engineering. These cloning experiments of *Qsd1* and *Qsd2* were conducted in different genetic backgrounds, and it remains to be determined whether these genes interact, which would require a collection of mutants in the same cultivar.

A *MAPKK3* gene was also identified as the causal gene of *Phs1* for grain dormancy in common wheat (Torada *et al*., 2016), based on a map‐based cloning approach using cvs. ‘Haruyokoi’ and ‘Leader’ with short and long dormancy, respectively. The introduction of the *Phs1* ‘Haruyokoi’ allele in the ‘Leader’ background led to relatively higher expression of the *TaMAPKK* gene in grains and shortened dormancy relative to that of ‘Leader’. These results indicated that the function of the *MAPKK3* gene for grain dormancy is conserved among the Triticeae. These findings are critical for breeding of pre‐harvest sprouting tolerance in barley and wheat.

Targeted mutagenesis via ‘genome editing’ by means of customizable endonucleases is a new breeding technology that can efficiently produce desired mutants in a target gene and has been applied to generate mutants in multiple crop species, including barley (Gerasimova *et al*., 2020; Kapusi *et al*., 2017; Lawrenson *et al*., 2015; Thiel *et al*., 2021). Genome editing makes it possible to create single or multiple mutations at the target site(s) of choice without any alteration in the genetic background, allowing for powerful analyses of the effects of individual genes and their genetic interactions. Currently, RNA‐guided clustered regularly interspaced short palindromic repeats (CRISPR)‐associated (Cas) endonucleases are the molecular tools of choice for targeted mutagenesis in cereal crops (Hisano *et al*., 2021; Koeppel *et al*., 2019). Higher‐order gene‐edited mutants can be generated at once, as demonstrated by the simultaneous targeting of all three *Qsd1* homeoalleles in the wheat ABD subgenomes, which produced prolonged grain dormancy (Abe *et al*., 2019). This result suggests that grain dormancy can be manipulated by deliberately introducing mutations. Here, we performed targeted mutagenesis of the barley *Qsd1* and *Qsd2* genes using RNA‐guided Cas9 endonuclease and analysed the consequences on dormancy in single and double mutants. The motivation for this study was fuelled by the need to fine‐tune barley grain dormancy for breeding by balancing pre‐harvest sprouting tolerance and uniform germination for malting.

## Results

### Generation and molecular analysis of *qsd1* and *qsd2* mutants in barley

**Figure 1:**
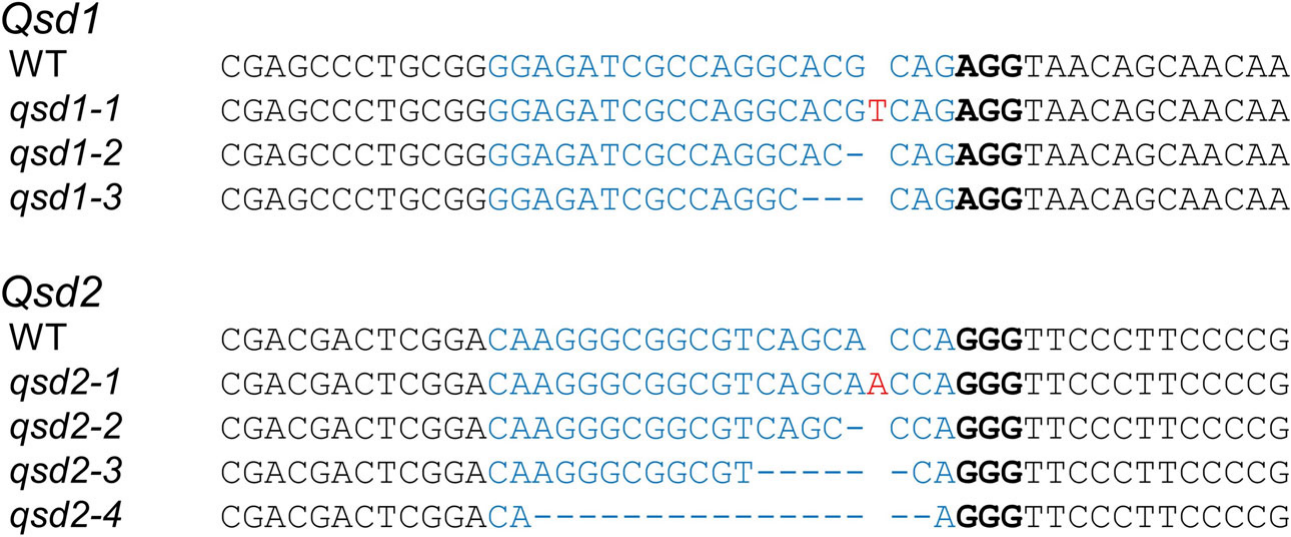
Comparison of DNA sequences for the Cas9/gRNA target sites in *Qsd1* and *Qsd2* in the wild type and mutants. Sanger sequencing was performed to detect mutations in *Qsd1* and *Qsd2*. Wild‐type, WT; *qsd1* mutants, *qsd1‐1* to *qsd1‐3*; *qsd2* mutants, *qsd2‐1* to *qsd2‐4*. *qsd1‐1*, 1‐bp insertion; *qsd1‐2*, 1‐bp deletion; *qsd1‐3*, 3‐bp deletion; *qsd2‐1*, 1‐bp insertion; *qsd2‐2*, 1‐bp deletion; *qsd2‐3*, 6‐bp deletion; *qsd2‐4*, 17‐bp deletion. These representative mutants were used for further analysis; other mutants are listed in Figure S1. The sequence targeted by the gRNA (protospacer) is shown in blue, the protospacer‐adjacent motif (PAM, bound by the Cas9 enzyme) is in bold, dashes indicate deletions, and red nucleotides indicate insertions.

We transformed ‘Golden Promise’ barley via *Agrobacterium tumefaciens*‐mediated DNA transfer to immature embryos using constructs expressing guide RNAs (gRNAs) designed against *Qsd1* or *Qsd2*. We obtained 21 and 70 hygromycin‐resistant T_0_ plants for *Qsd1* and *Qsd2*, respectively (Table 1). After PCR amplification of the gRNA target regions and Sanger sequencing, we identified nine and 21 independently mutated plants for *Qsd1* and *Qsd2*, respectively (Figure 1, Figure S1). The obtained *qsd1* alleles featured 1‐ to 4‐bp deletions or 1‐bp insertions (Figure S1). Likewise, *qsd2* alleles comprised one 1‐bp insertion event, small deletions from 1 to 7 bp, and a slightly larger deletion of 17 bp (Figure S1). We did not observe any base substitutions in the plants analysed. We allowed T_0_ plants to self‐pollinate and collected M_2_ grains (T_1_ generation). We excised immature embryos from these grains and put them on the culture medium for precocious germination to shorten generation time.

**Table 1:** Summary of targeted mutagenesis of *Qsd1* and *Qsd2* genes in barley

target gene	No. of regenerated plants	No. of mutated plants	Efficiency of mutation events per regenerated plants (%)
*Qsd1*	21	9	42.9
*Qsd2*	70	21	30.0

We tested for segregation of gene‐edited alleles at *Qsd1* and *Qsd2* by Sanger sequencing of the M_2_ generation. In addition, we determined whether individual plants harboured the T‐DNA by PCR on genomic DNA (Figure S2). We thus selected T‐DNA‐free and homozygous *qsd1* mutants with a 1‐bp insertion (*qsd1‐1*), a 1‐bp deletion (*qsd1‐2*), or a 3‐bp deletion (*qsd1‐3*) and *qsd2* mutants with a 1‐bp insertion (*qsd2‐1*), a 1‐bp deletion (*qsd2‐2*), a 6‐bp deletion (*qsd2‐3*), and a 17‐bp deletion (*qsd2‐4*) for further analysis (Figure 1). The deduced amino acid sequences of Qsd1 and Qsd2 from the wild‐type and the mutants are shown in Figure S3. While wild‐type Qsd1 contains 494 amino acids, the protein is shortened by one amino acid in *qsd1‐3*; the other mutants carry premature stop codons that lead to truncated proteins of 72 (*qsd1‐1*) or 67 (*qsd1‐2*) amino acids. Similarly, wild‐type Qsd2 is a 523‐amino acid protein that is shorter by two amino acids in *qsd2‐3* and is truncated to 42 (*qsd2‐1*), 46 (*qsd2‐2*), or 36 (*qsd2‐4*) amino acids.

### Grain dormancy tests in M_3_ (T_2_ generation) lines

**Figure 2:**
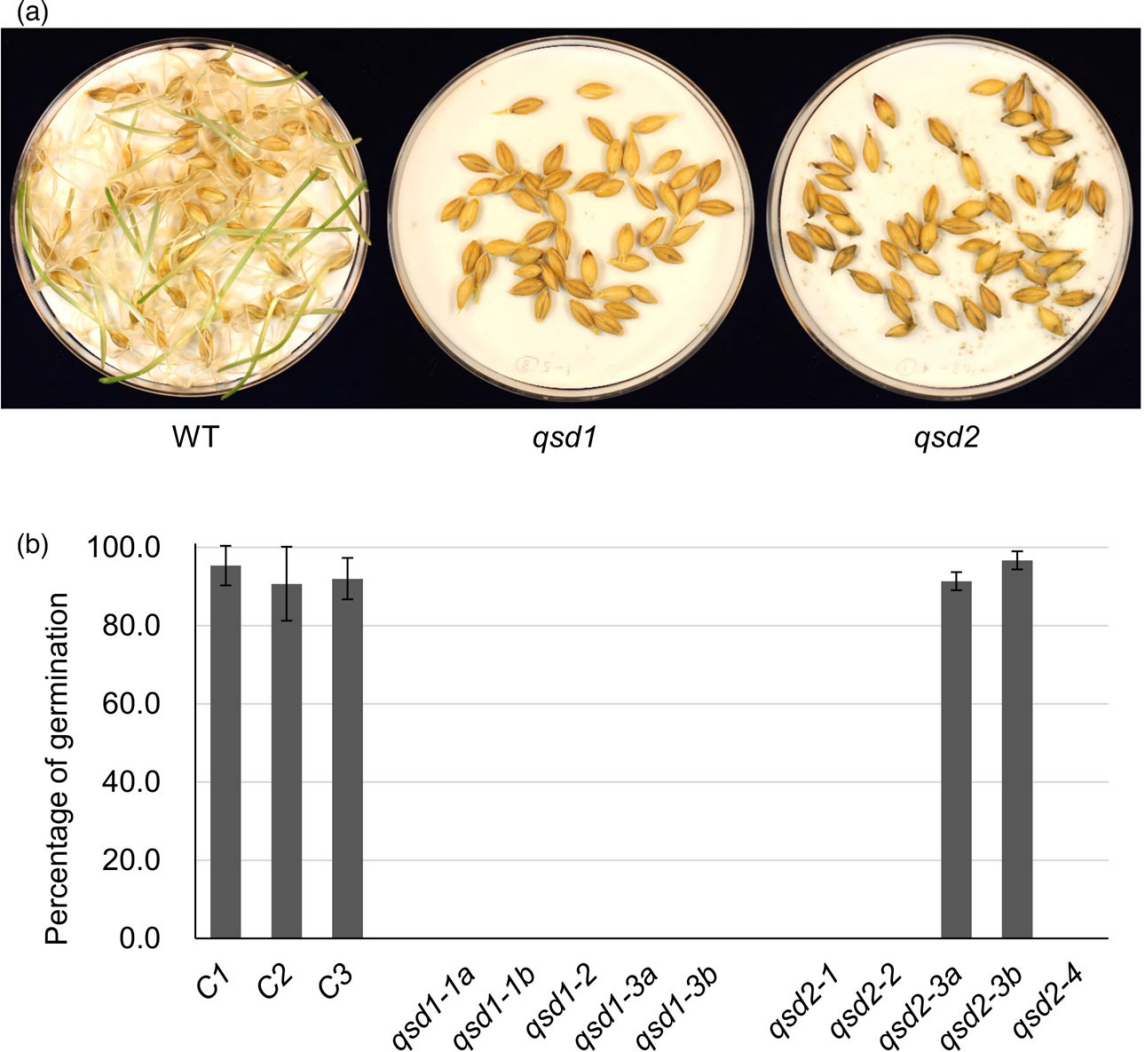
Loss of *Qsd1* or *Qsd2* function results in prolonged dormancy in barley. Germination test on wild‐type (WT) and genome‐edited barley *qsd1* and *qsd2* mutant grains. All grains were after‐ripened at 25 °C for 6 weeks under dry conditions to break dormancy. (a) Representation photographs of WT and mutant grains 7 days after grain imbibition. (b) Mean percentages of germination in wild‐type and mutant lines. Data are shown as means ± standard deviation (SD) from three replicates of 50 grains each. C1; non‐transgenic wild‐type Golden Promise control; C2 and C3, non‐edited control plants segregated from T_0_ plants for *Qsd1* and *Qsd2*, respectively. *qsd1‐1a*, *qsd1‐1b*, *qsd1‐3a*, *qsd1‐3b*, *qsd2‐3a*, and *qsd2‐3b* were derived from individual M_2_ plants.

We next determined grain dormancy in the wild type and mutants. The level of dormancy was evaluated as percentage of germination within a given period of time, where a shorter dormancy causes a higher percentage of germination. We used M_2_ progeny from T_0_ plants without a T‐DNA and with the wild‐type *Qsd1* and *Qsd2* alleles as controls. We performed germination tests on grains that had been after‐ripened for 6 weeks at 25 °C, which revealed that all controls started to germinate within 1 day after grain imbibition; almost all grains had germinated by 4 days (Figure S4). Seven days after grain imbibition, the controls as well as the in‐frame mutant *qsd2‐3* displayed over 90% germination proportion (Figure 2), indicating that this in‐frame mutation does neither disrupt nor decrease Qsd2 function. By contrast, all other *qsd1* and *qsd2* mutants, including the in‐frame mutant *qsd1‐3*, did not germinate during the 7 days after grain imbibition (Figure 2). However, grains did germinate after treatment with 3% hydrogen peroxide (Figure S5), indicating that these mutant grains exhibit extremely long dormancy rather than grain lethality.

### Generating and phenotyping of *qsd1* and *qsd2* double mutants

**Figure 3:**
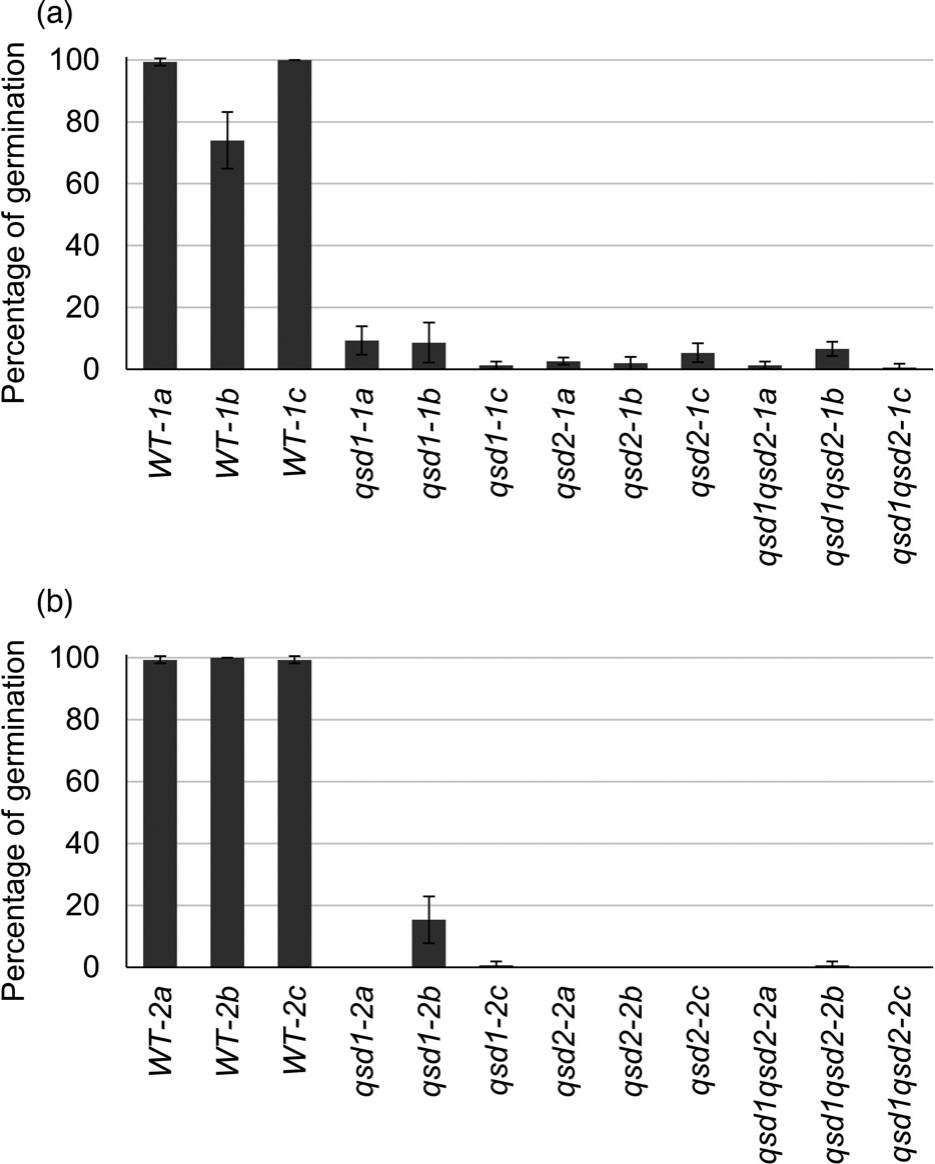
Percentage of germination of F_3_ lines harbouring the *qsd1* and/or *qsd2* mutations 21 days after grain imbibition. Percentage of germination of F_3_ lines resulting from crosses between *qsd2‐4* as a seed parent and *qsd1‐1* (a) or *qsd1‐3* (b) as the pollen donors. All grains were after‐ripened at 25 °C for 6 weeks under dry conditions and an additional 4 weeks at 40 °C to reduce dormancy. Data are shown as means ± standard deviation (SD) from three replicates of 50 grains each. *qsd1* mutant lines: *qsd1‐1x*, *qsd1‐2x*; *qsd2* mutant lines: *qsd2‐1x*, *qsd2‐2x*; *qsd1qsd2* double mutant lines: *qsd1qsd2‐1x*, *qsd1qsd2‐2x*; wild‐type siblings: WT‐1x, WT‐2x.

We generated two independent *qsd1* and *qsd2* double mutants by crossing a *qsd2* mutant (*qsd2‐4*) with two mutants carrying different *qsd1* mutant alleles (*qsd1‐1* and *qsd1‐3*). We selected four genotypes of T‐DNA‐free F_3_ lines that were either wild‐type for *Qsd1* and *Qsd2*, homozygous for *qsd1* or *qsd2* (single mutants), or homozygous for *qsd1* and *qsd2* (double mutant). The representative results of PCR analysis to check the presence or absence of the T‐DNA in F_2_ plants are shown in Figure S2. These results allowed us to confirm that the T‐DNA had been removed. Representative F_2_ plants are shown in Figure S6: we observed no obvious differences in their growth or flowering time. We then scored percentage of germination for all F_3_ lines after 6 weeks of after‐ripening at 25 °C and an additional 4 weeks at 40 °C. While wild‐type grains appeared to fully germinate within 7 days after grain imbibition, none of the single or double mutant lines did (Figure S7). A more quantitative analysis revealed that the controls germinated at proportions between 70% and close to 100% at 21 days after grain imbibition, whereas all single and double mutants showed similarly low germination proportions, with values spanning 1.3%–9.3% (for *qsd1*), 2%–5.3% (for *qsd2*), and 0.6%–6.7% (for *qsd1qsd2*) for the *qsd2‐4*×*qsd1‐1* progeny; and 0%–15.3% (for *qsd1*), 0% (for *qsd2*), and 0%–0.7% (for *qsd1qsd2*) for the *qsd2‐4*×*qsd1‐3* progeny (Figure 3).

We also performed pre‐harvest sprouting tests using five unthreshed mature spikes of F_3_ progenies derived from *qsd2‐4*×*qsd1‐1*. All wild‐type spikes sprouted, but the *qsd1*, *qsd2*, and *qsd1qsd2* double mutants did not sprout at all (Figure S8).

### Germination of mutants at low temperature

**Figure 4:**
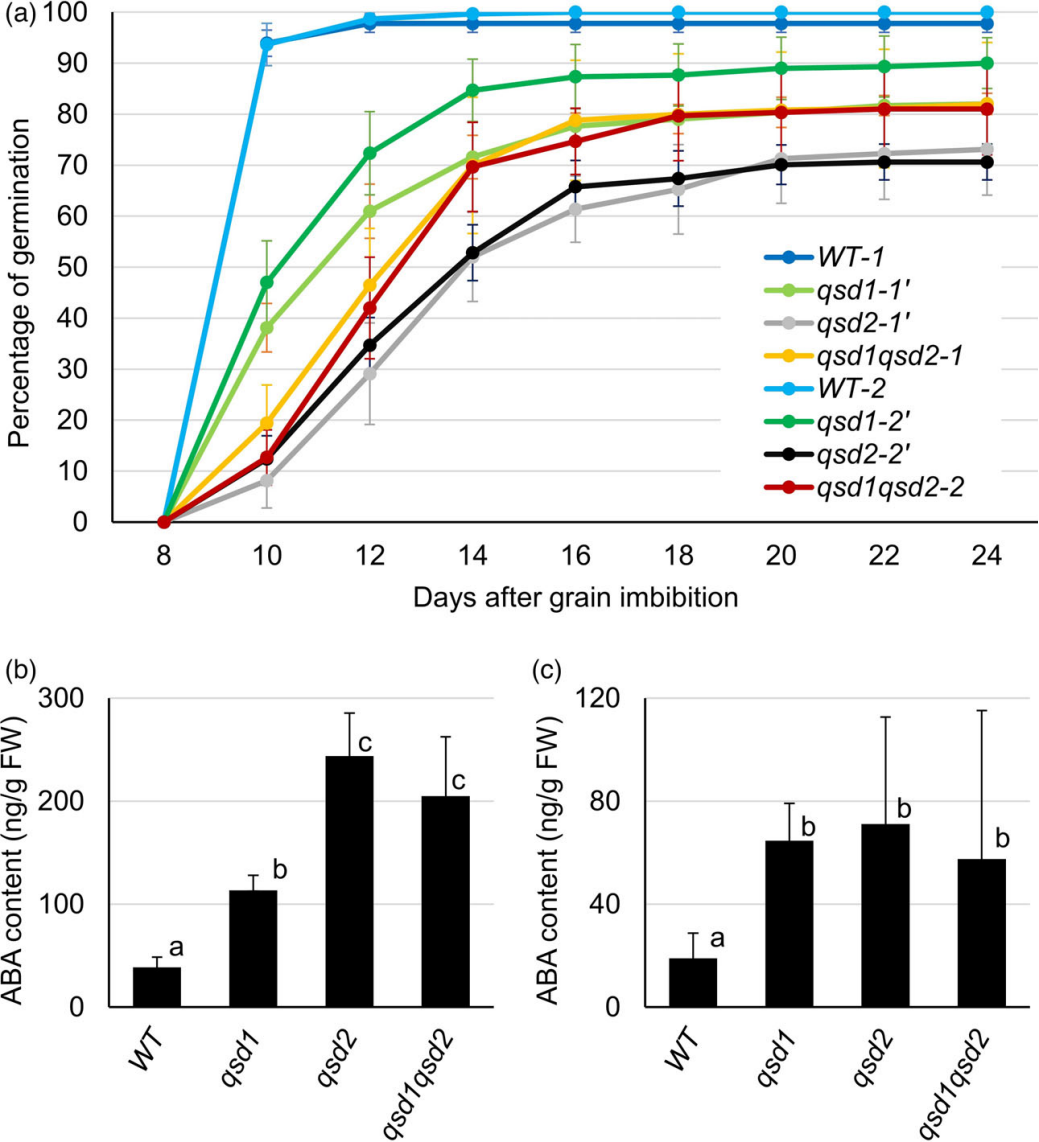
Germinations and ABA contents of F_3_ lines harbouring the *qsd1* and/or *qsd2* mutations at 4 °C. All grains were after‐ripened at 25 °C for 6 weeks under dry conditions and an additional 4 weeks at 40 °C to reduce dormancy. Grains were allowed to germinate at 4 °C. (a) Time course of percentages of germination from eight to 24 days after grain imbibition. Data are shown as means ± standard error (SE) from six replicates of 21–50 grains each. *qsd1* mutant lines: *qsd1‐1*′, *qsd1‐2*′; *qsd2* mutant lines: *qsd2‐1*′ *qsd2‐2*′; *qsd1qsd2* double mutant lines: *qsd1qsd2‐1*, *qsd1qsd2‐2*; wild‐type siblings: WT‐1, WT‐2. *qsd1‐1*′, *qsd2‐1*′, *qsd1qsd2‐1* and WT‐1 are segregating siblings derived from the *qsd2‐4*×*qsd1‐1* progeny, and *qsd1‐2*′, *qsd2‐2*′, *qsd1qsd2‐2* and WT‐2 are those from the *qsd2‐4*×*qsd1‐3* progeny. (b, c) ABA content in embryos during germination at 4 °C 3 days (b) or seven days (c) after grain imbibition, respectively. Data are shown as means ± SD from six replicates. Different letters indicate significant differences, as determined by Tukey’s test (*P* < 0.05).

As with other plants, grain dormancy in barley is alleviated by exposure to low temperature. We thus scored germination percentages of *qsd1* and *qsd2* grains maintained at 4 °C and in the dark from the start of grain imbibition. None of the genotypes germinated during the 8 days (Figure 4a). However, by 10 days after grain imbibition, wild‐type grains had reached over 90% germination, whereas the mutants exhibited delayed germination, as evidenced by their lower germination proportions: 38%–47% for *qsd1*, 6%–13% for *qsd2*, and 12%–20% for *qsd1qsd2*. Germination further increased slightly 12 days after grain imbibition, with proportions of 60%–73% (*qsd1*), 29%–35% (*qsd2*), and 42%–47% (*qsd1qsd2*). Twenty days after imbibition, *qsd1* and *qsd2* mutants showed 80%–90% and 70%–72% germination proportions, respectively. Notably, the *qsd1qsd2* double mutants had a higher percentage of germination, at 81%–82%, compared with the *qsd2* single mutant.

We concluded that exposure to low temperature following grain imbibition largely abrogates dormancy in mutant grains, although with a slight delay in the mutants compared to the wild type. To investigate the possible reason for this delay, we measured ABA contents in embryos after grain imbibition but before germination took place. ABA content was significantly higher in embryos from the *qsd1* mutant compared with wild‐type embryos 3 days after grain imbibition, and with *qsd2* and *qsd1qsd2* embryos accumulating over twice as much ABA as the *qsd1* mutant (*P* < 0.001, Figure 4b). Seven days after grain imbibition, ABA content was lower in all genotypes relative to levels measured at 3 days (Figures 4b,c). In addition, wild‐type embryos accumulated significantly less ABA than the mutants that had comparable ABA contents of approximately three times that of the wild type (*P* < 0.05, Figure 4c).

### Germination test under different light conditions

**Figure 5:**
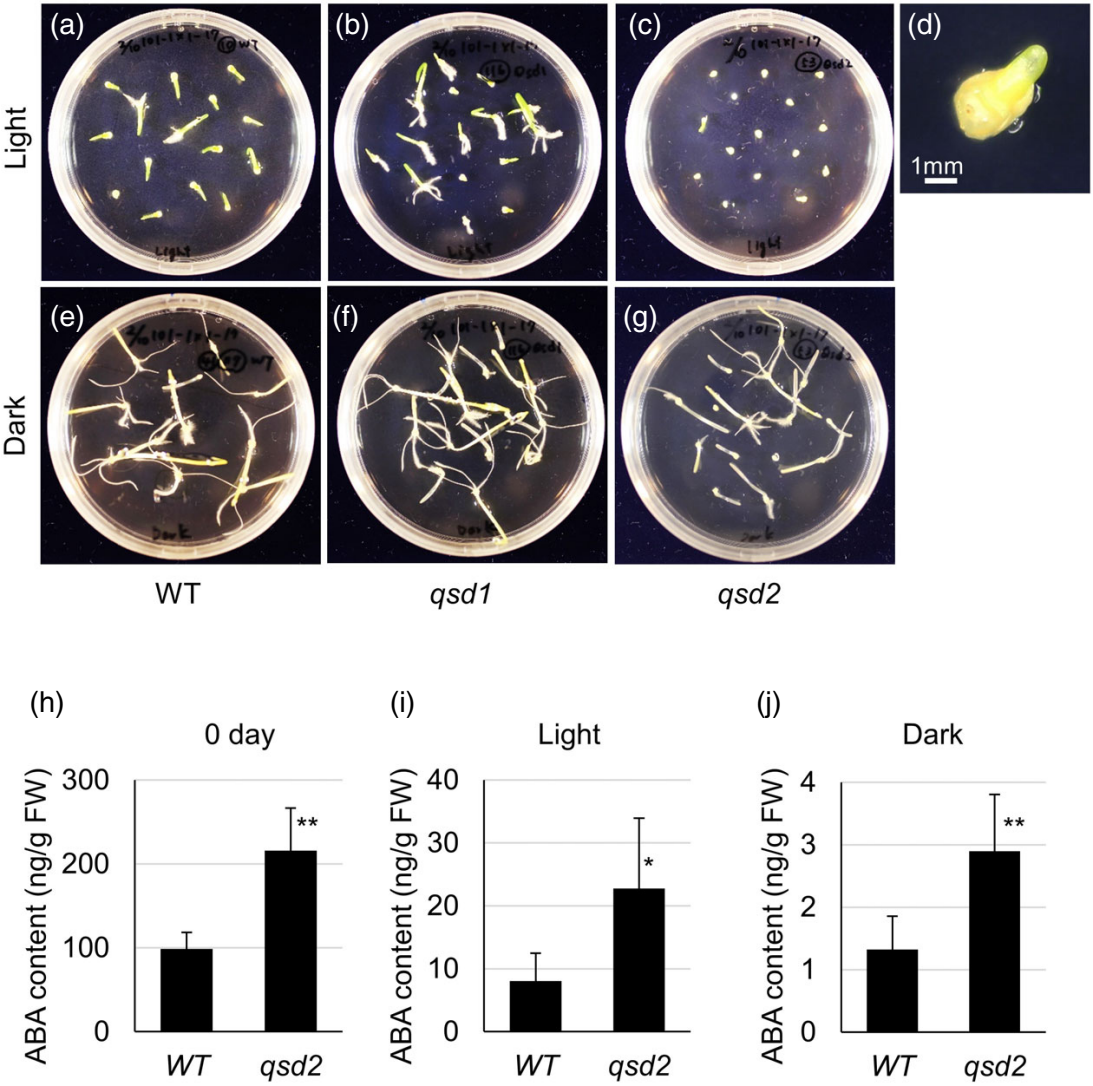
Germination of immature embryos incubated in the dark or in the light. Germination tests in the light (a–d) or in the dark (e–g) at 25 °C were conducted using immature embryos of the wild‐type (WT) (a, e), *qsd1* (b, f), and *qsd2* (c, d, g) mutants. Plates were photographed 7 days after the start of culture. d is a magnified view of a stagnating *qsd2* embryo after 10 days in the light. (h–j) ABA contents in immature WT and *qsd2* embryos at the start of the experiment (0 days, h) and after 7 days in the light (i) or in the dark (j). Statistical analysis was performed by Student's *t*‐test between the WT and *qsd2*. Asterisks indicate significant differences (*P < 0.05, **P < 0.01).

Exposure to light also affects grain dormancy and germination. We therefore explored the effect of light exposure on germination using an immature embryo germination system, thus removing any influence from the endosperm and husks. Here, we considered an immature embryo as having germinated when the primary shoot and root grew more than 5 mm in length. Over 90% of immature embryos produced shoots and roots, regardless of the genotype, when immature embryos were incubated in the dark (Figure 5e–g, Table 2). In sharp contrast, *qsd2* and *qsd1qsd2* immature embryos rarely germinated when placed in the light; wild‐type and *qsd1* immature embryos only exhibited a modest suppression of germination under the same conditions (Figure 5a–d, Table 2).

**Table 2:** Germination proportion of barley immature embryos incubated in the dark or in the light

	Light			Dark		
	*n*	Ave*	SD^†^	*n*	Ave*	SD^†^
wild type	4	15.2^a^	4.9	4	18.7^a^	1.2
*qsd1*	4	13.7^a^	2.8	4	18.4^a^	1.1
*qsd2*	4	4.9^b^	3.1	4	18.1^a^	1.3
*qsd1qsd2*	3	5.7^b^	1.0	3	18.4^a^	1.5

* Average of germination number per 20 immature embryos on the embryo culture medium at 25 °C. Different characters indicate significant differences (*P* < 0.05) as analysed by Tukey's test.

† SD means standard deviation.

We finally measured the ABA content of immature embryos and cultured embryos. Immature embryos in *qsd2* accumulated twice as much ABA as the wild type (*P* < 0.01, Figure 5h). Seven days after initiation of culture, the ABA content of both wild‐type and *qsd2* cultured embryos had dropped by 90% in the light and by over 98% in the dark. Notably, *qsd2* still had higher ABA levels than the wild type (*P* < 0.05, Figure 5i,j).

## Discussion

Genetic variation in genes related to grain dormancy or germination are essential resources for breeding of pre‐harvest sprouting tolerance. Many QTL analyses have been performed for grain dormancy in barley, highlighting the two major QTL, *Qsd1* and *Qsd2* (Nakamura *et al*., 2016; Sato *et al*., 2016). However, each QTL and associated causal gene was characterized in different genetic backgrounds, making any comparison of their respective effects, or their interactions, difficult to interpret. Here, we employed Cas9‐mediated genome editing to specifically target the *Qsd1* and *Qsd2* loci in the same genetic background of the barley cultivar ‘Golden Promise’ (Figure 1, Figure S1, Table 1). Grains from these mutants exhibited long dormancy but were nonetheless viable (Figures 2 and 3, Figure S5). This prolonged dormancy must also contribute to tolerance for pre‐harvest sprouting (Figure S8). Our results suggest that targeted mutagenesis of *Qsd1* and *Qsd2* is a useful strategy to prolong grain dormancy in barley. This is the first report of controlling germination of barley grains using targeted mutagenesis.

The use of Cas9‐mediated gene editing was previously reported in barley, for instance, by targeting *HvPM19*, an ABA‐inducible gene that encodes a plasma membrane protein predicted to affect grain dormancy. However, no phenotypic characterization was conducted (Lawrenson *et al*., 2015). Our results show that mutations in *Qsd1* and *Qsd2* result in prolonged grain dormancy compared with wild‐type ‘Golden Promise’, observations that are in line with results previously obtained by RNAi‐mediated suppression of *Qsd1* in barley cv. ‘Golden Promise’ and EMS mutants for *Qsd2* in barley cv. ‘Barke’ (Nakamura *et al*., 2016; Sato *et al*., 2016). Like the *qsd1* mutants, the *Qsd1*‐RNAi transformants showed little germination in almost the same environment as used in the present study (Sato *et al*., 2016). The EMS‐induced *Qsd2* mutant has a different genetic background and the environmental conditions and methods used for its analysis were different from those used in our current work, so it is not possible to make an exact comparison, but the EMS mutant seems to have moderately prolonged dormancy compared with our *qsd2* mutants in this study (Nakamura *et al*., 2016). We hypothesize that barley cultivation and breeding have selected alleles with enhanced function at *Qsd1* and *Qsd2* to promote rapid germination, from the pool of natural alleles represented among barley cultivars (Nakamura *et al*., 2016; Sato *et al*., 2016; Vetch *et al*., 2020). We attempted to break dormancy by using the high temperature of 40 °C for 4 weeks, with a dormant after‐ripening period of 10 weeks; however, even under these extreme conditions, the germination of all mutants was still significant delayed at 25 °C, thus preventing a comparative analysis of the individual contribution of *Qsd1* and *Qsd2* to dormancy (Figure 3). We therefore suspect that both genes are essential for germination under natural settings and, conversely, that loss‐of‐function alleles cause severely delayed germination and are therefore under strong selective pressure. This hypothesis should be tested on plants grown in the field, but the level of grain dormancy is expected to fluctuate due to temperature, wind, rain, and pests and diseases in a natural environment. We have conducted all of the current experiments in an environmentally controlled growth chamber, to exclude the possibility that the extent of dormancy might be shortened in barley grown in the field particularly due to higher temperatures that plants would experience during the ripening period. Mimicking natural growth conditions may help dissect the individual contributions of *Qsd1* and *Qsd2* to dormancy.

In barley breeding, rapid germination has been selected for in malting barley cultivars, while long dormancy confers tolerance to pre‐harvest sprouting. Creating weak mutant alleles is considered an effective strategy to modulate traits more subtly than loss‐of‐function alleles, for example, by targeting the 3′ end of genes or to delete just one or a few amino acids while retaining the translational reading frame. Gene editing systems with Cas endonucleases will allow such precise genomic modifications (Komor *et al*., 2016; Nishida *et al*., 2016; Schedel *et al*., 2017). In this study, we identified one in‐frame mutation each for *Qsd1* (*qsd1‐3*) and *Qsd2* (*qsd2‐3*), resulting in the removal of one (*qsd1‐3*) or two (*qsd2‐3*) amino acids near the N terminus of the proteins. Dormancy in *qsd2‐3* was not affected, in contrast to all other *qsd2* mutants isolated in this study. Thus two‐amino‐acid deletion may not alter the kinase activity of Qsd2, as its kinase domain is located between amino acids 100 and 300 (Figure S3) (Nakamura *et al*., 2016). Although the *qsd1‐3* allele encodes a protein lacking a single amino acid, a histidine residue at position 32, this mutant showed prolonged dormancy. This suggests that *qsd1‐3* is a loss‐of‐function mutation of *Qsd1*. The deletion of a histidine residue may have affected the secondary structure of Qsd1, leading to an inactive protein. We performed the F_3_ experiments assuming an in‐frame mutant allele would be milder for grain dormancy; however, the results were similar compared with frame‐shifted mutations. How these proteins perform their function for grain dormancy and germination remains to be investigated. The mutants produced in this study are in the same genetic background and will be instrumental in understanding these issues.

Under low‐temperature (4 °C) conditions, the *qsd2* mutants exhibited a significant delay of germination than did *qsd1* mutants (Figure 4). This observation suggests that the dormancy or germination defect in *qsd2* might be stronger than that of *qsd1* in the ‘Golden Promise’ genetic background. On the other hand, the germination percentage of the *qsd2* single mutant was lower than that of the *qsd1qsd2* double mutant and *qsd1* single mutant. It cannot be ruled out that the *qsd1* mutation might counteract the suppressive effect of *qsd2* on germination under cold conditions, although the underlying molecular mechanism is unknown.

We also performed germination assays using cultured immature embryos incubated in the dark or in the light (Figure 5a–g, Table 2). In the dark, wild‐type and mutant immature embryos showed the same germination proportion. In sharp contrast, *qsd2* mutants exhibited a strong suppression of germination when incubated in the light. The CRYPTOCHROME blue light photoreceptors perceive light and repress germination in barley grains (Barrero *et al*., 2014). Loss of Qsd1 did not affect embryo germination in the light, indicating that Qsd1 acts independently from light signalling related to germination or dormancy of barley grains. By contrast, germination of *qsd2* mutant immature embryos was repressed by light. *Qsd2* encodes MAPKK3, suggesting that MAPK cascades might be involved in photoreception during germination of barley grains. A phosphoproteomic analysis of freshly harvested and after‐ripened barley embryos treated with ABA revealed the involvement of different phosphorylation signalling networks in each set of embryos, suggesting that after‐ripening modulates phosphorylation signalling pathways, leading to the decay of ABA signalling (Ishikawa *et al*., 2019). Although it remains unclear whether Qsd2 is a key MAPKK in this phosphorylation pathway linked to ABA regulation, we did notice an effect of *qsd2* mutants on ABA accumulation in immature embryos (Figure 5).

Although different plant species have different dormancy mechanisms, reflecting their physiology and morphology (Baskin and Baskin, 2004), *Arabidopsis* (*Arabidopsis thaliana*) and barley share core conserved mechanisms. For example, nitric oxide can break dormancy in both *Arabidopsis* and barley (Bethke *et al*., 2004), as do GA and ROS. The cytochrome P450 gene *ABA 8ʹ‐HYDROXYLASE* is involved in ABA catabolism and thus regulates dormancy or germination in both *Arabidopsis* and barley (Millar *et al*., 2006). When measuring ABA contents, we discovered that ABA accumulates to higher levels in the mutants compared to the wild type (Figures 4b,c, 5h–j). The *qsd2* mutants initially had higher ABA levels than *qsd1* mutants or the wild type 3 days after grain imbibition, but these levels dropped to become comparable to those of *qsd1* mutants after 7 days at 4 °C (Figure 5h–j). Likewise, both *qsd1* and *qsd2* mutants germinated later than the wild type under cold conditions, suggesting that while ABA may delay the initial germination in the mutants, it does not contribute to long‐term dormancy of the barley grain. In addition, ABA levels in *qsd2* immature embryos were higher than those of wild‐type embryos after 7 days of incubation in the light or in the dark; notably, ABA levels in dark‐incubated *qsd2* embryos reached only ~2% of non‐cultured *qsd2* embryos, suggesting the possibility that such ABA content allowed germination in *qsd2*. Although Wang *et al*. (1995) reported that ABA contents are higher in dormant barley embryos compared with non‐dormant embryos, they concluded that ABA content is not related to grain germination. Further detailed analysis is needed to clarify whether ABA directly affects germination in the *qsd2* mutants.

Genes involved in seed dormancy have been isolated from forward genetic studies and studies of natural variation in *Arabidopsis* and other species. *DELAY OF GERMINATION 1* (*DOG1*) was isolated from a major QTL for seed dormancy in *Arabidopsis* accessions, with the *dog1* mutant having reduced dormancy (Bentsink *et al*., 2006). *DOG1* is expressed at higher levels in *Arabidopsis* seeds ripened at low temperatures (Kendall *et al*., 2011). DOG1 physically interacts with the protein phosphatases ABA HYPERSENSITIVE GERMINATION 1 (AHG1) and AHG3 (Nishimura *et al*., 2018); however, the function of DOG1 has not fully been elucidated. *DOG1*‐like genes have been identified in barley and wheat, and surprisingly they exhibited distinct expression patterns from *Arabidopsis DOG1* (Ashikawa *et al*., 2010). Although these genes share low sequence identity with *Arabidopsis DOG1*, their ectopic overexpression in *Arabidopsis* demonstrated their conserved function in seed dormancy. In another example, the rice *Seed dormancy 4* (*Sdr4*) QTL was identified by map‐based cloning using near isogenic lines derived from crosses between the short dormancy *japonica*‐type rice cv. ‘Nipponbare’ and long dormancy *indica*‐type rice cv. ‘Kasalath’ (Sugimoto *et al*., 2010). *Sdr4* expression was positively controlled by *VIVIPAROUS‐1* (*VP1*), the rice ortholog to *Arabidopsis ABA INSENSITIVE 3* (*ABI3*), a master regulator of seed dormancy as well as seed maturation (Hattori *et al*., 1994; McCarty *et al*., 1991). A rice *sdr4* mutant was also shown to be insensitive to ABA, that is the germination of the mutant was not blocked by ABA, although ABA does block germination of wild‐type grains. Rice *Sdr4* is therefore thought to play an intermediate regulator role in dormancy during grain maturation; in agreement, the positive regulators of germination *GA20 OXIDASE 1* (*OsGA20ox‐1*) and *PLASMA MEMBRANE INTRINSIC PROTEIN 1;3* (*PIP1;3*) were highly expressed in the *sdr4* mutant after grain imbibition. The loss of *DOG1* and *Sdr4* functions were associated with shortened dormancy (Bentsink *et al*., 2006; Sugimoto *et al*., 2010). On the other hand, loss of *Qsd1* and *Qsd2* might result in tolerance to pre‐harvest sprouting due to longer dormancy. However, too long of a dormancy period could be difficult for malting. So, the use of multiple genes for natural variation of seed/grain dormancy and their precise control will be required for future breeding.

In this study, we used targeted mutagenesis to introduce mutations in the barley genes *Qsd1* and *Qsd2*, in which loss of function resulted in prolonged grain dormancy. The introduction of mutations in the same genetic background further allowed us to investigate their genetic interactions. *qsd2* dominated dormancy responses slightly more than *qsd1*, although *qsd1* did appear to mitigate the effect of *qsd2* on suppressing germination under cold conditions. For a practical use for barley breeding, we had hoped for mutants with shorter dormancy, but all mutants showed long dormancy phenotypes. However, this experiment demonstrated that grain dormancy can be regulated by mutations in *Qsd1* and *Qsd2*, paving the way for the creation of additional alleles by genome editing, for instance by the use of base editors like artificial deaminases (Nishida *et al*., 2016).

## Experimental procedures

### Plant materials

Barley cv. ‘Golden Promise’ was grown in growth chambers for 2 months in daily cycles of 12 h light at 15 °C and 12 h darkness at 13 °C before transfer to a long‐day photoperiod (16 h light at 16 °C and 8 h darkness at 13 °C) until flowering (ca. 1 month in long days) to collect immature embryos for *Agrobacterium*‐mediated transformation. Transgenic and mutant barley plants were grown in growth chambers in long days (16 h light at 16 °C and 8 h darkness at 13 °C).

### Design of guide RNAs and vector construction

Guide RNAs (gRNAs) were designed to target each exon of barley *Qsd1* and *Qsd2* with the gRNA online design tool WU‐CRISPR (http://crisprdb.org/wu‐crispr/; Wong *et al*., 2015). The predicted secondary structures of gRNAs were generated by RNAfold (http://rna.tbi.univie.ac.at/cgi‐bin/RNAWebSuite/RNAfold.cgi), and the most suitable targets were selected largely considering the criteria described by Liang *et al*. (2016) and Kumlehn *et al*. (2018). Consequently, Qsd1‐gRNA1 (5′‐GGAGATCGCCAGGCACGCAG‐3′) and Qsd2‐gRNA1 (5′‐AAGGGCGGCGTCAGCACCA‐3′) were chosen for this study (Figure S9).

The synthetic oligonucleotides (5′‐tggcGGAGATCGCCAGGCACGCAG‐3′ and 5′‐aaacCTGCGTGCCTGGCGATCTCC‐3′ for *Qsd1*, 5′‐tggcAAGGGCGGCGTCAGCACCA‐3′ and 5′‐aaacTGGTGCTGACGCCGCCCTT‐3′ for *Qsd2*) were annealed and ligated between the *OsU3* promoter and the gRNA scaffold in the intermediate vector pSH121 (Gerasimova *et al*., 2018) predigested with the restriction enzyme *Bsa*I. The lowercase sequences in the synthetic oligonucleotides are the overhang sticky ends for ligation with *Bsa*I‐cleaved plasmid DNA. The expression units for gRNAs and Cas9 were introduced into the binary vector p6i‐2×35S‐TE9 (DNA Cloning Service e.K., Germany) using the *Sfi*I restriction sites. The T‐DNA of p6i‐2×35S‐TE9 confers resistance to hygromycin in plants. The components of the T‐DNA region of the final vector used for transformation are depicted in Figure S10.

### Production and molecular analysis of transgenic barley plants

Transformation vectors were introduced into *Agrobacterium* strain AGL1. The resulting agrobacteria were then used for transformation via co‐cultivation with barley immature embryos as previously described (Hisano *et al*., 2017; Hisano and Sato, 2016).

Total DNA was extracted from regenerated plantlets with Kaneka Easy DNA Extraction Kit version 2 (Kaneka, Japan) and used as a template for PCR validation of the presence of transgenes; PCR products spanning the genomic region targeted by each gRNA were PCR‐amplified and sequenced to identify mutations. PCR was performed following the method described by Hisano and Sato (2016). Mutations were detected by visual inspection of sequencing data from PCR products using the ‘Assemble Sequence to Reference’ module in CLC Main Workbench software (QIAGEN, Germany). The primers used in this study are listed in Table S1.

### Immature embryo culture

For rapid generation and to prevent germination delays due to dormancy, immature embryo culture was performed on half‐strength Murashige and Skoog (MS) medium adjusted to pH 5.8, with 15 g/L sucrose and 3 g/L Phytagel (Sigma‐Aldrich). To this end, immature embryos were isolated from immature grains, ca. 20 days after pollination, that had been surface sterilized with a solution of sodium hypochlorite (1% effective chlorine concentration), and incubated on the above growth medium for 2 days in the dark and for 2–3 days under long days (16 h light/8 h dark) at 25 °C. Germinated embryos were then transferred to a growth chamber and sown in soil. To analyse the response of embryo germination to light, immature embryos were incubated in the dark or exposed to light in a long‐day photoperiod under fluorescent lights (17.7 μmol m^−2^ s^−1^ PPF in 400–500 nm blue region of the spectrum, and 53.0 μmol m^−2^ s^−1^ PPF in 400–700 nm) at 25 °C.

### Generation of double mutants

Double mutants were generated by conventional crossing of an M_2_ mutant line carrying a 17‐bp deletion in *Qsd2* with two independent M_2_ mutant lines with either a 1‐bp insertion or a 3‐bp deletion in *Qsd1*.

### Grain germination test

The spikes of each plant were harvested at physiological maturity when the colour of rachis had changed to a straw yellow. Spikes were desiccated at 25 °C for 10 days in a drying cabinet (Tolihan Co., Japan) and then stored at –20 °C until all samples were collected. The spikes of M_2_ plants were hand‐threshed at the same time, and M_3_ grains were after‐ripened at 25 °C and 10%–15% relative humidity for 6 weeks. Spikes from F_2_ plants were treated similarly, with an additional 4 weeks at 40 °C after 6 weeks of after‐ripening at 25 °C. Fifty grains were assessed for germination on moistened filter paper (ADVANTEC, Japan) in 90‐mm disposable dishes with triplication at 25 °C for normal germination tests. For low‐temperature response germination tests at 4 °C, 21–50 grains per dish with six replications were used. The percentage of germination was calculated by counting seedlings with primary shoots and roots that had elongated more than 5 mm.

For pre‐harvest sprouting tests, unthreshed mature spikes of F_3_ progenies of *qsd2‐4*×*qsd1‐1* were maintained on wet soil for 11 days with daily cycles of 16 h light at 25 °C and 8 h darkness at 15 °C.

### Analysis of ABA content

Embryos from four or five grains for each sample representing 11–33 mg fresh weight were collected with five replicates and lyophilized in a freeze dryer. The embryos were sampled at 3 days or 7 days after grain imbibition for the test under 4 °C conditions, and at 7 days after the start of incubation for the test under light/dark conditions. Five immature embryos were collected from each spike as controls. Dried embryos were then ground and used for extraction and analysis of phytohormones according to Hisano *et al*. (2016) and Tsukahara *et al*. (2015).

## Conflict of interest

The authors declare no conflicts of interest associated with this manuscript.

## Author contributions

HH, FA, SN, JK, and KS designed experiments, HH, RH, HM, and TM performed experiments, ME, MM, and SN provided vectors, sequences of target genes and supported experiments, HH, FA, SN, JK, and KS drafted the manuscript, and RH, ME, and MM revised the manuscript. All authors looked through the final version of the manuscript and approved the submission.

## Supporting information


**Figure S1** Partial DNA sequence of the gRNA target sites at the *Qsd1* and *Qsd2* loci in mutants.
**Figure S2** PCR analysis for detection of T‐DNA region in T_1_ and T‐DNA‐free mutant plants.
**Figure S3** Partial deduced amino acid sequences from the wild‐type and *qsd1* and *qsd2* mutants.
**Figure S4**. Germination of the wild‐type and M_3_ lines of genome‐edited barley 4 days after grain imbibition.
**Figure S5** Germination of the M_3_ *qsd1‐2* mutant 1 month after treatment with 3% hydrogen peroxide.
**Figure S6** T‐DNA‐free F_2_ generation of wild‐type, *qsd1* and *qsd2* single mutant, and *qsd1qsd2* double mutant plants.
**Figure S7** Germination from segregating progeny with no mutation in *Qsd1* or *Qsd2* (wild‐type) or homozygous for *qsd1*, *qsd2*, or *qsd1qsd2* double mutations (photographed 7 days after grain imbibition).
**Figure S8** Pre‐harvest sprouting test of F_3_ progenies derived from *qsd2‐4*×*qsd1‐1* (photographed 11 days after place spikes on the soil).
**Figure S9** Target positions and 20‐nt sequence of gRNAs in *Qsd1* and *Qsd2* genes.
**Figure S10** Structure of the T‐DNA region in the vector used in this study.
**Table S1**. PCR primers used in this study
