# Supplementary material for: Regulation of germination by targeted mutagenesis of grain dormancy genes in barley
Source: Plant Biotechnol J. 2021 Sep 8;20(1):37–46. doi: 10.1111/pbi.13692 (PMC8710902; doi:10.1111/pbi.13692)
Supplement: Supplementary file 1 — Figure S1 Partial DNA sequence of the gRNA target sites at the Qsd1 and Qsd2 loci in mutants. Figure S2 PCR analysis for detection of T‐DNA region in T1 and T‐DNA‐free mutant plants. Figure S3 Partial deduced amino acid sequences from the wild‐type and qsd1 and qsd2 mutants. Figure S4. Germination of the wild‐type and M3 lines of genome‐edited barley 4 days after grain imbibition. Figure S5 Germination of the M3 qsd1‐2 mutant 1 month after treatment with 3% hydrogen peroxide. Figure S6 T‐DNA‐free F2 generation of wild‐type, qsd1 and qsd2 single mutant, and qsd1qsd2 double mutant plants. Figure S7 Germination from segregating progeny with no mutation in Qsd1 or Qsd2 (wild‐type) or homozygous for qsd1, qsd2, or qsd1qsd2 double mutations (photographed 7 days after grain imbibition). Figure S8 Pre‐harvest sprouting test of F3 progenies derived from qsd2‐4×qsd1‐1 (photographed 11 days after place spikes on the soil). Figure S9 Target positions and 20‐nt sequence of gRNAs in Qsd1 and Qsd2 genes. Figure S10 Structure of the T‐DNA region in the vector used in this study. Table S1. PCR primers used in this study [file PBI-20-37-s001.pptx]

## Slide 1
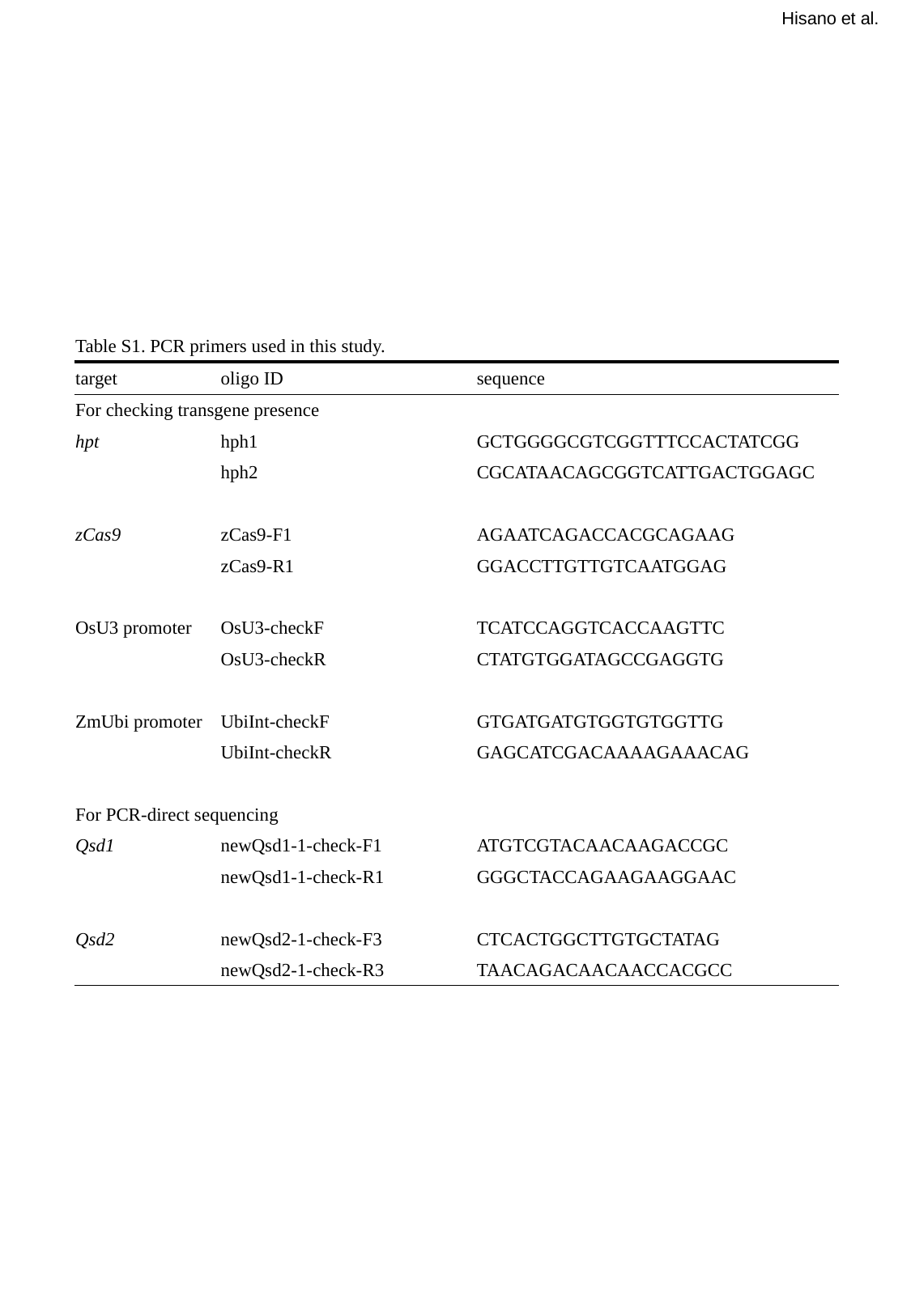

Hisano et al.
| Table S1. PCR primers used in this study. | | |
| --- | --- | --- |
| target | oligo ID | sequence |
| For checking transgene presence | | |
| hpt | hph1 | GCTGGGGCGTCGGTTTCCACTATCGG |
| | hph2 | CGCATAACAGCGGTCATTGACTGGAGC |
| | | |
| zCas9 | zCas9-F1 | AGAATCAGACCACGCAGAAG |
| | zCas9-R1 | GGACCTTGTTGTCAATGGAG |
| | | |
| OsU3 promoter | OsU3-checkF | TCATCCAGGTCACCAAGTTC |
| | OsU3-checkR | CTATGTGGATAGCCGAGGTG |
| | | |
| ZmUbi promoter | UbiInt-checkF | GTGATGATGTGGTGTGGTTG |
| | UbiInt-checkR | GAGCATCGACAAAAGAAACAG |
| | | |
| For PCR-direct sequencing | | |
| Qsd1 | newQsd1-1-check-F1 | ATGTCGTACAACAAGACCGC |
| | newQsd1-1-check-R1 | GGGCTACCAGAAGAAGGAAC |
| | | |
| Qsd2 | newQsd2-1-check-F3 | CTCACTGGCTTGTGCTATAG |
| | newQsd2-1-check-R3 | TAACAGACAACAACCACGCC |

## Slide 2
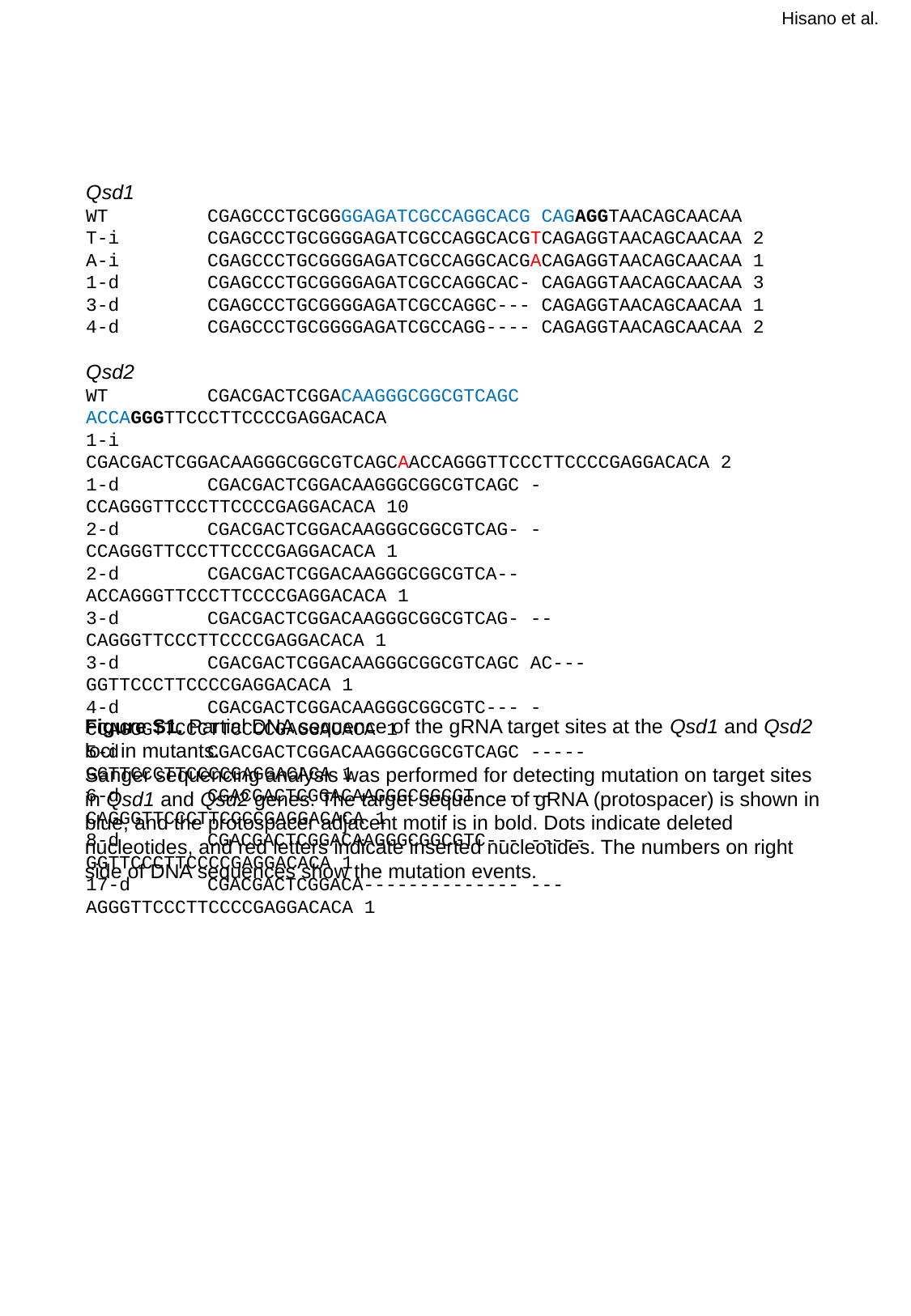

Hisano et al.
Qsd1
WT	CGAGCCCTGCGGGGAGATCGCCAGGCACG CAGAGGTAACAGCAACAA
T-i	CGAGCCCTGCGGGGAGATCGCCAGGCACGTCAGAGGTAACAGCAACAA 2
A-i	CGAGCCCTGCGGGGAGATCGCCAGGCACGACAGAGGTAACAGCAACAA 1
1-d	CGAGCCCTGCGGGGAGATCGCCAGGCAC- CAGAGGTAACAGCAACAA 3
3-d	CGAGCCCTGCGGGGAGATCGCCAGGC--- CAGAGGTAACAGCAACAA 1
4-d	CGAGCCCTGCGGGGAGATCGCCAGG---- CAGAGGTAACAGCAACAA 2
Qsd2
WT	CGACGACTCGGACAAGGGCGGCGTCAGC ACCAGGGTTCCCTTCCCCGAGGACACA
1-i	CGACGACTCGGACAAGGGCGGCGTCAGCAACCAGGGTTCCCTTCCCCGAGGACACA 2
1-d	CGACGACTCGGACAAGGGCGGCGTCAGC -CCAGGGTTCCCTTCCCCGAGGACACA 10
2-d	CGACGACTCGGACAAGGGCGGCGTCAG- -CCAGGGTTCCCTTCCCCGAGGACACA 1
2-d	CGACGACTCGGACAAGGGCGGCGTCA-- ACCAGGGTTCCCTTCCCCGAGGACACA 1
3-d	CGACGACTCGGACAAGGGCGGCGTCAG- --CAGGGTTCCCTTCCCCGAGGACACA 1
3-d	CGACGACTCGGACAAGGGCGGCGTCAGC AC---GGTTCCCTTCCCCGAGGACACA 1
4-d	CGACGACTCGGACAAGGGCGGCGTC--- -CCAGGGTTCCCTTCCCCGAGGACACA 1
5-d	CGACGACTCGGACAAGGGCGGCGTCAGC -----GGTTCCCTTCCCCGAGGACACA 1
6-d	CGACGACTCGGACAAGGGCGGCGT---- --CAGGGTTCCCTTCCCCGAGGACACA 1
8-d	CGACGACTCGGACAAGGGCGGCGTC--- -----GGTTCCCTTCCCCGAGGACACA 1
17-d	CGACGACTCGGACA-------------- ---AGGGTTCCCTTCCCCGAGGACACA 1
Figure S1. Partial DNA sequence of the gRNA target sites at the Qsd1 and Qsd2 loci in mutants.
Sanger sequencing analysis was performed for detecting mutation on target sites in Qsd1 and Qsd2 genes. The target sequence of gRNA (protospacer) is shown in blue, and the protospacer adjacent motif is in bold. Dots indicate deleted nucleotides, and red letters indicate inserted nucleotides. The numbers on right side of DNA sequences show the mutation events.

## Slide 3
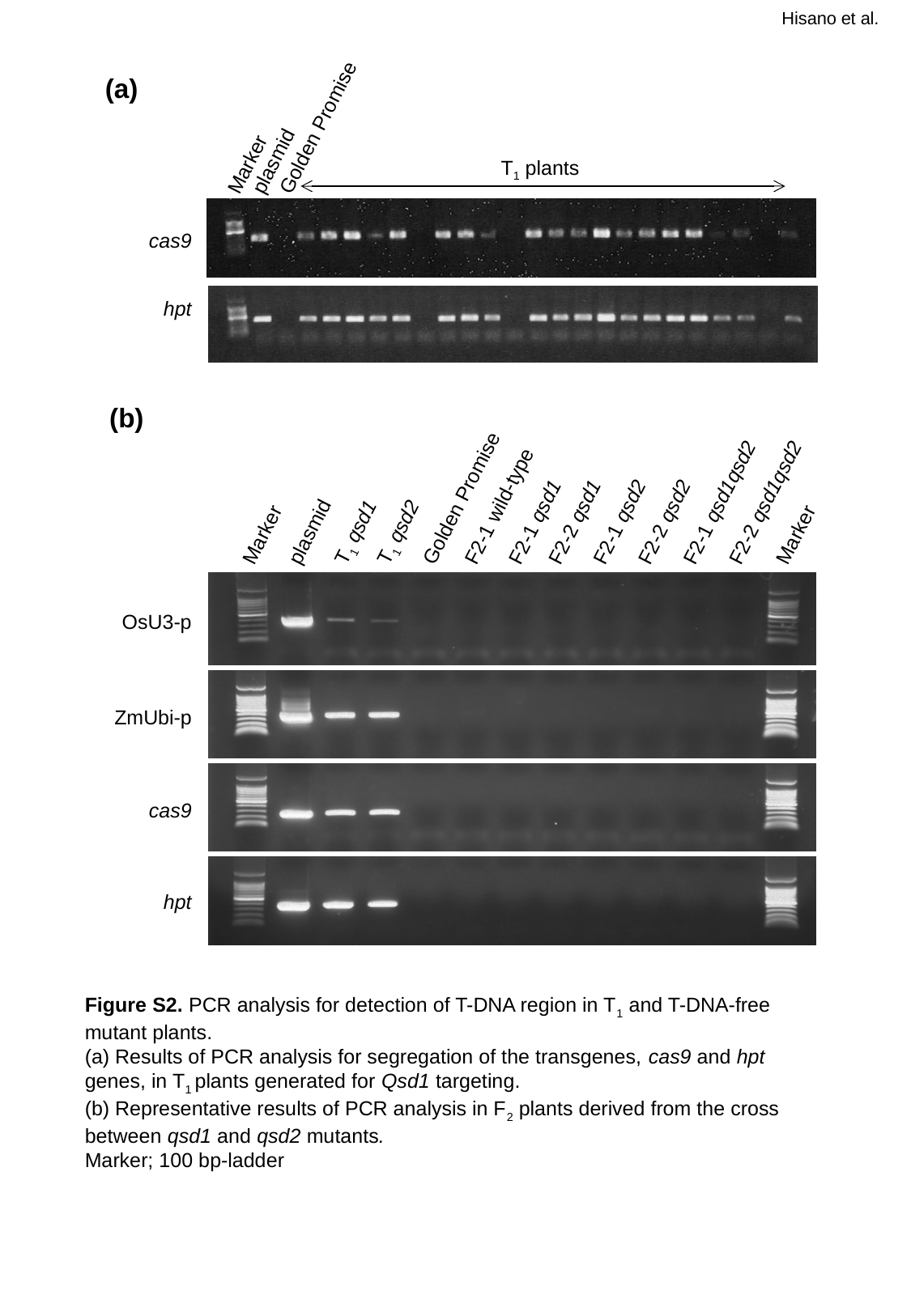

Hisano et al.
(a)
Golden Promise
Marker
plasmid
T1 plants
cas9
hpt
(b)
Golden Promise
F2-2 qsd1qsd2
F2-1 wild-type
F2-1 qsd1qsd2
F2-1 qsd1
F2-2 qsd1
F2-1 qsd2
F2-2 qsd2
Marker
plasmid
T1 qsd1
T1 qsd2
Marker
OsU3-p
ZmUbi-p
cas9
hpt
Figure S2. PCR analysis for detection of T-DNA region in T1 and T-DNA-free mutant plants.
(a) Results of PCR analysis for segregation of the transgenes, cas9 and hpt genes, in T1 plants generated for Qsd1 targeting.
(b) Representative results of PCR analysis in F2 plants derived from the cross between qsd1 and qsd2 mutants.
Marker; 100 bp-ladder

## Slide 4
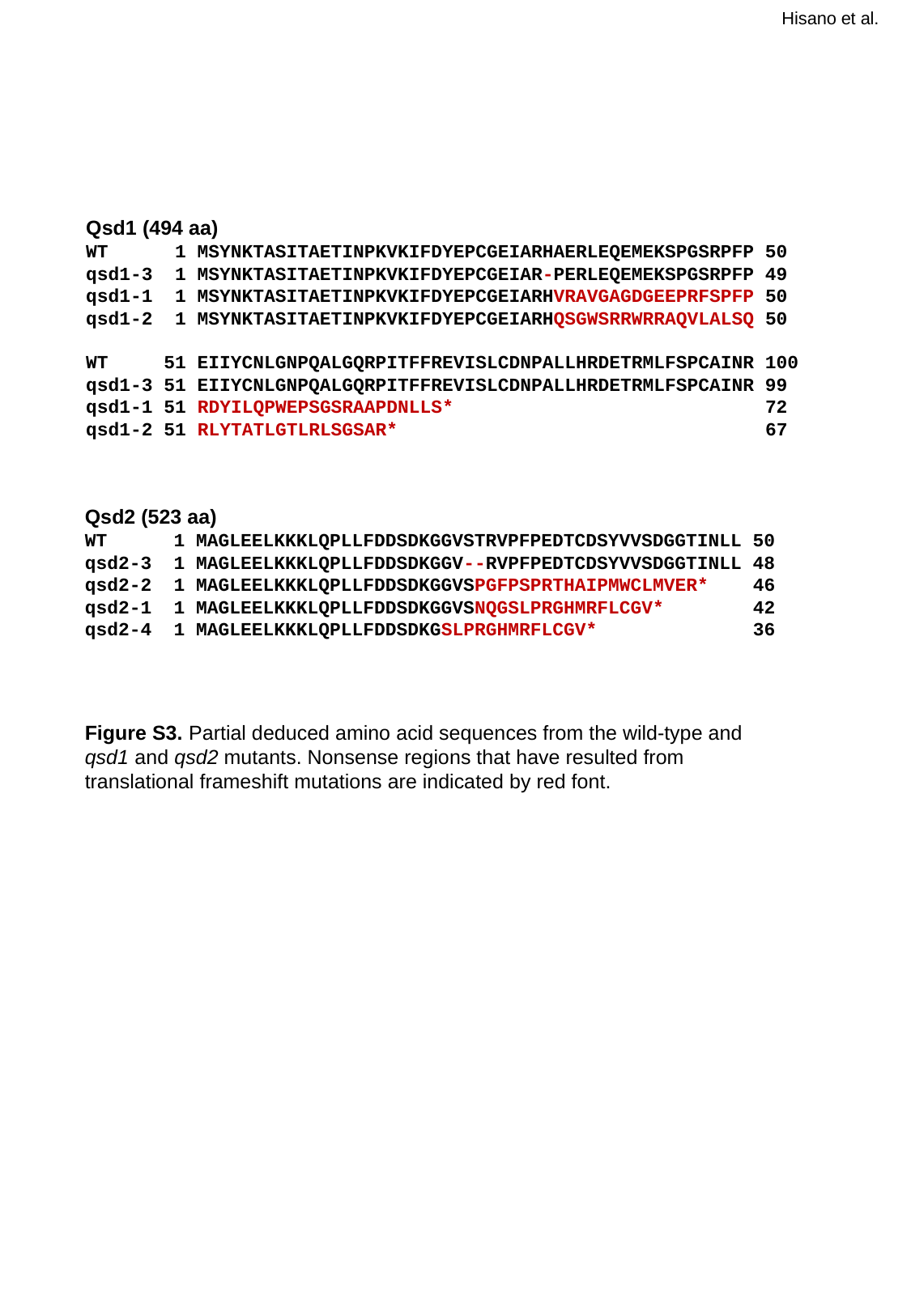

Hisano et al.
Qsd1 (494 aa)
WT 1 MSYNKTASITAETINPKVKIFDYEPCGEIARHAERLEQEMEKSPGSRPFP 50
qsd1-3 1 MSYNKTASITAETINPKVKIFDYEPCGEIAR-PERLEQEMEKSPGSRPFP 49
qsd1-1 1 MSYNKTASITAETINPKVKIFDYEPCGEIARHVRAVGAGDGEEPRFSPFP 50
qsd1-2 1 MSYNKTASITAETINPKVKIFDYEPCGEIARHQSGWSRRWRRAQVLALSQ 50
WT 51 EIIYCNLGNPQALGQRPITFFREVISLCDNPALLHRDETRMLFSPCAINR 100
qsd1-3 51 EIIYCNLGNPQALGQRPITFFREVISLCDNPALLHRDETRMLFSPCAINR 99
qsd1-1 51 RDYILQPWEPSGSRAAPDNLLS* 72
qsd1-2 51 RLYTATLGTLRLSGSAR* 67
Qsd2 (523 aa)
WT 1 MAGLEELKKKLQPLLFDDSDKGGVSTRVPFPEDTCDSYVVSDGGTINLL 50
qsd2-3 1 MAGLEELKKKLQPLLFDDSDKGGV--RVPFPEDTCDSYVVSDGGTINLL 48
qsd2-2 1 MAGLEELKKKLQPLLFDDSDKGGVSPGFPSPRTHAIPMWCLMVER* 46
qsd2-1 1 MAGLEELKKKLQPLLFDDSDKGGVSNQGSLPRGHMRFLCGV* 42
qsd2-4 1 MAGLEELKKKLQPLLFDDSDKGSLPRGHMRFLCGV* 36
Figure S3. Partial deduced amino acid sequences from the wild-type and qsd1 and qsd2 mutants. Nonsense regions that have resulted from translational frameshift mutations are indicated by red font.

## Slide 5
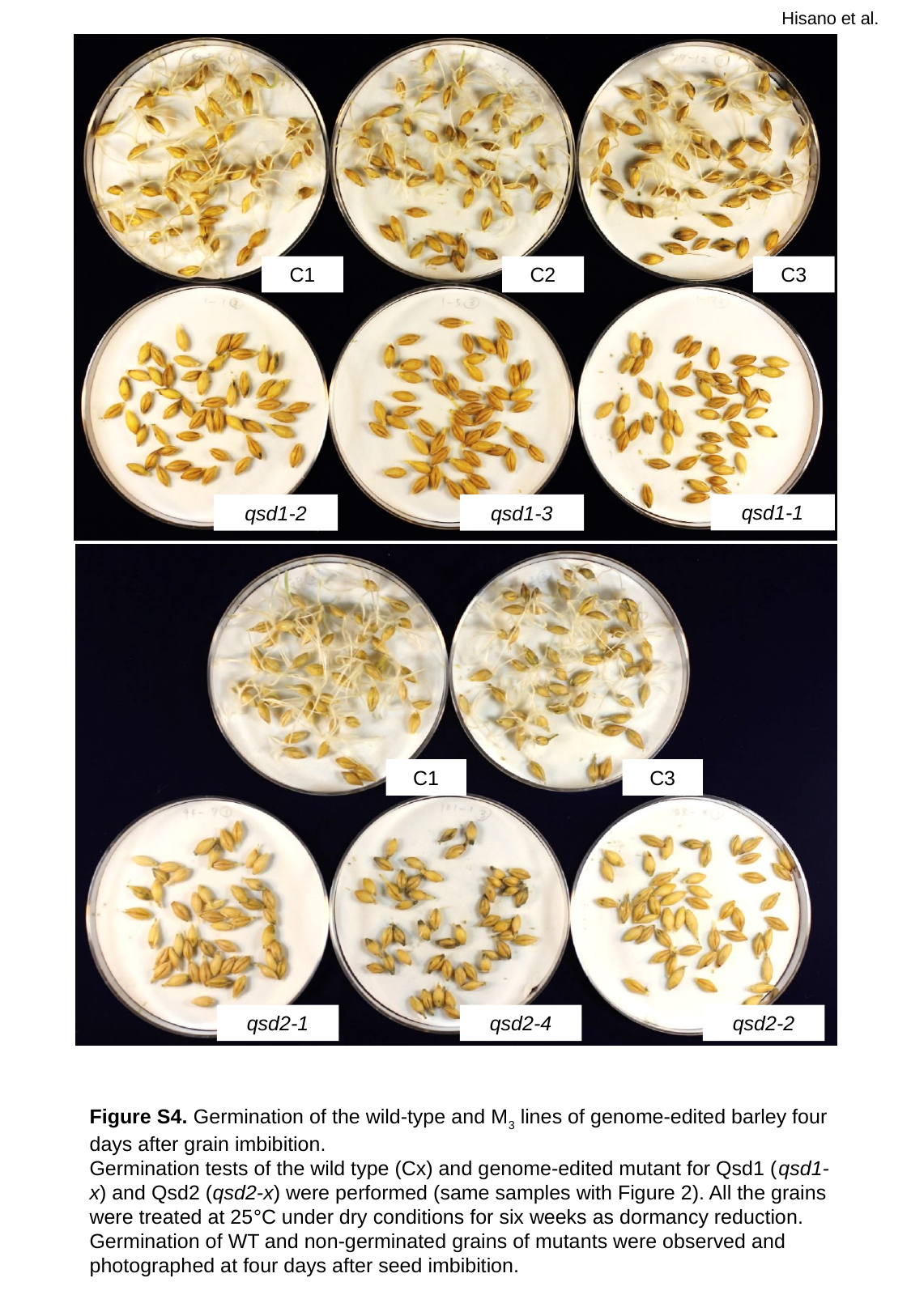

Hisano et al.
C1
C2
C3
qsd1-1
qsd1-2
qsd1-3
C1
C3
qsd2-1
qsd2-4
qsd2-2
Figure S4. Germination of the wild-type and M3 lines of genome-edited barley four days after grain imbibition.
Germination tests of the wild type (Cx) and genome-edited mutant for Qsd1 (qsd1-x) and Qsd2 (qsd2-x) were performed (same samples with Figure 2). All the grains were treated at 25°C under dry conditions for six weeks as dormancy reduction. Germination of WT and non-germinated grains of mutants were observed and photographed at four days after seed imbibition.

## Slide 6
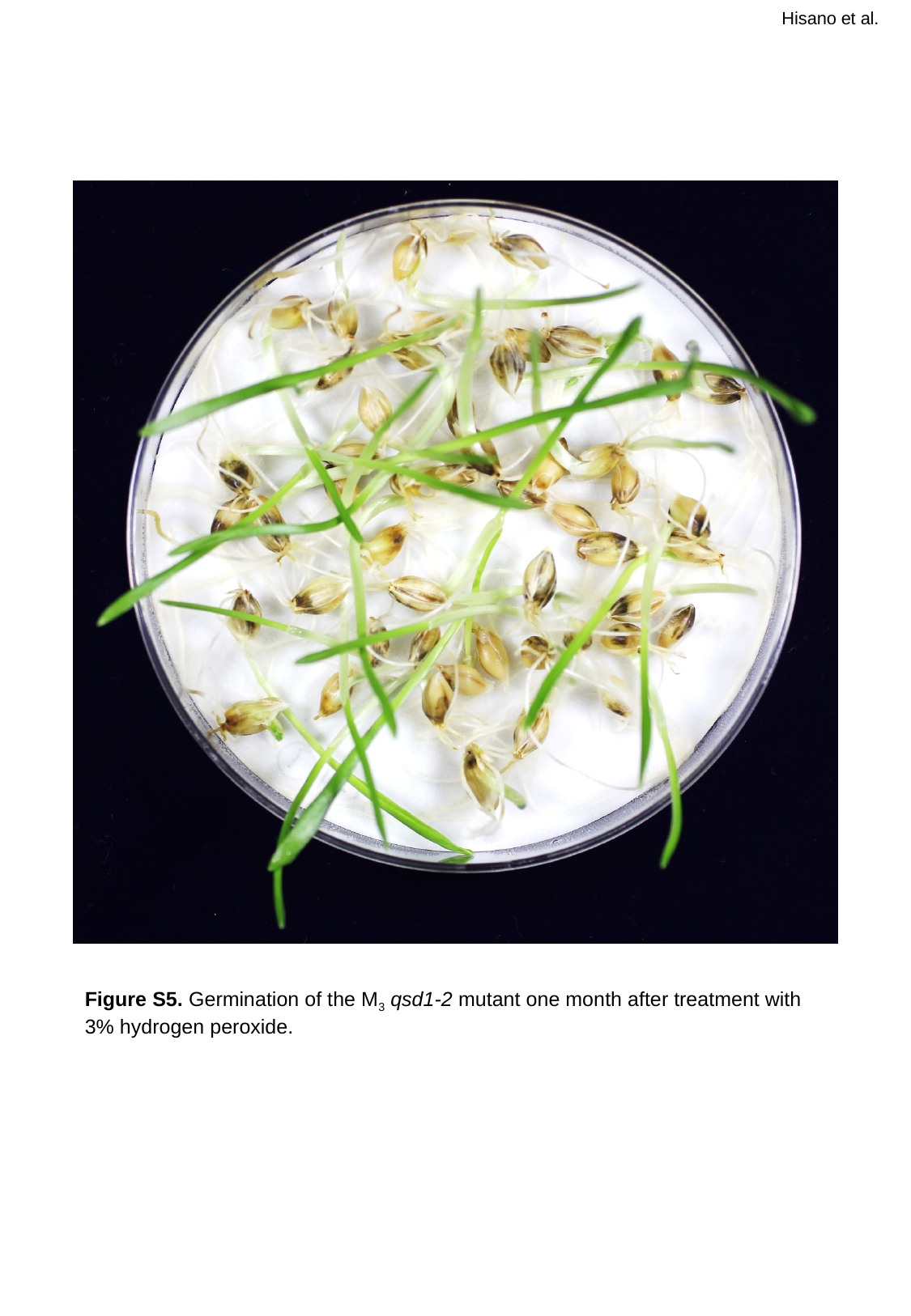

Hisano et al.
Figure S5. Germination of the M3 qsd1-2 mutant one month after treatment with 3% hydrogen peroxide.

## Slide 7
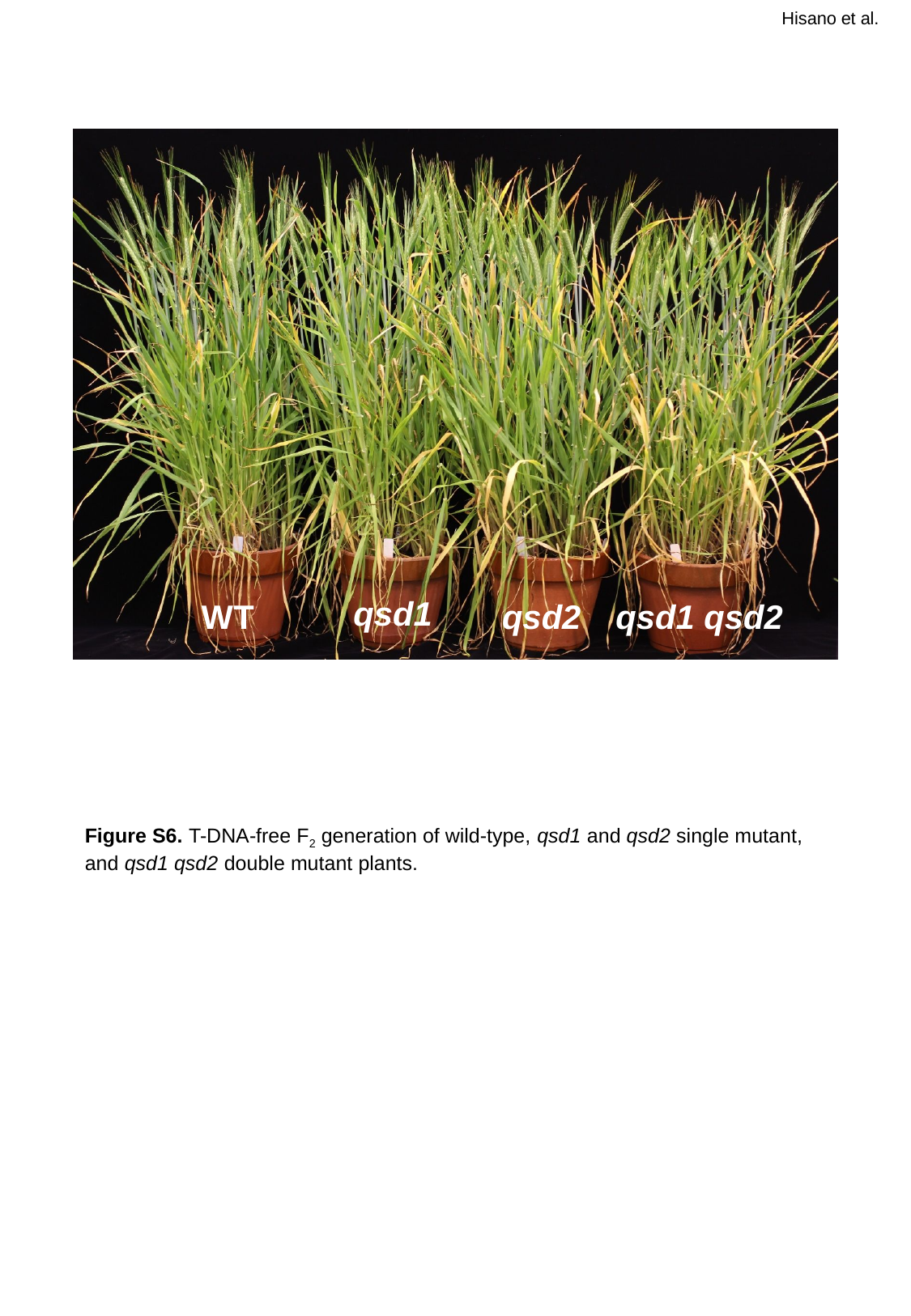

Hisano et al.
qsd1
WT
qsd2
qsd1 qsd2
Figure S6. T-DNA-free F2 generation of wild-type, qsd1 and qsd2 single mutant, and qsd1 qsd2 double mutant plants.

## Slide 8
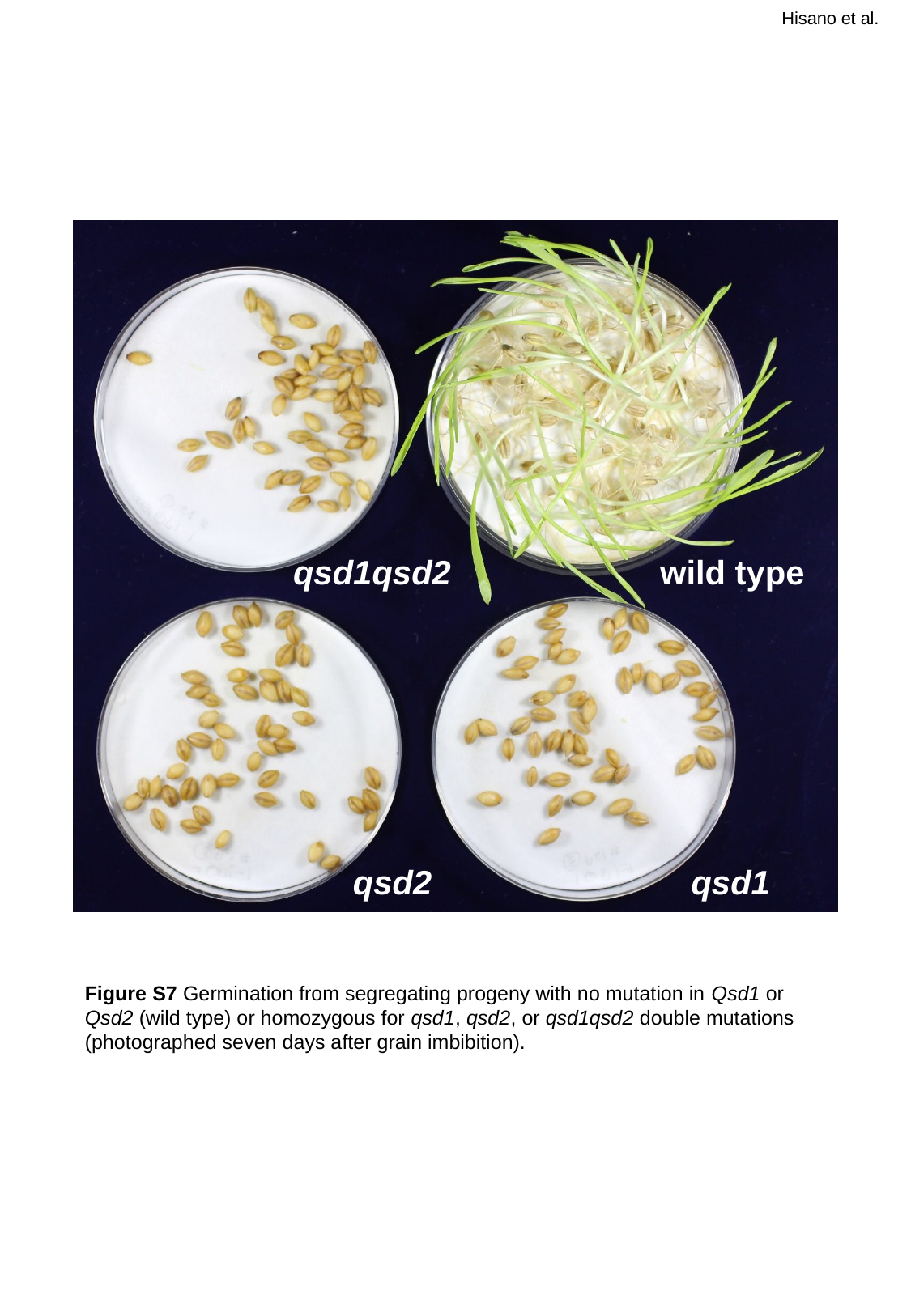

Hisano et al.
qsd1qsd2
wild type
qsd2
qsd1
Figure S7 Germination from segregating progeny with no mutation in Qsd1 or Qsd2 (wild type) or homozygous for qsd1, qsd2, or qsd1qsd2 double mutations (photographed seven days after grain imbibition).

## Slide 9
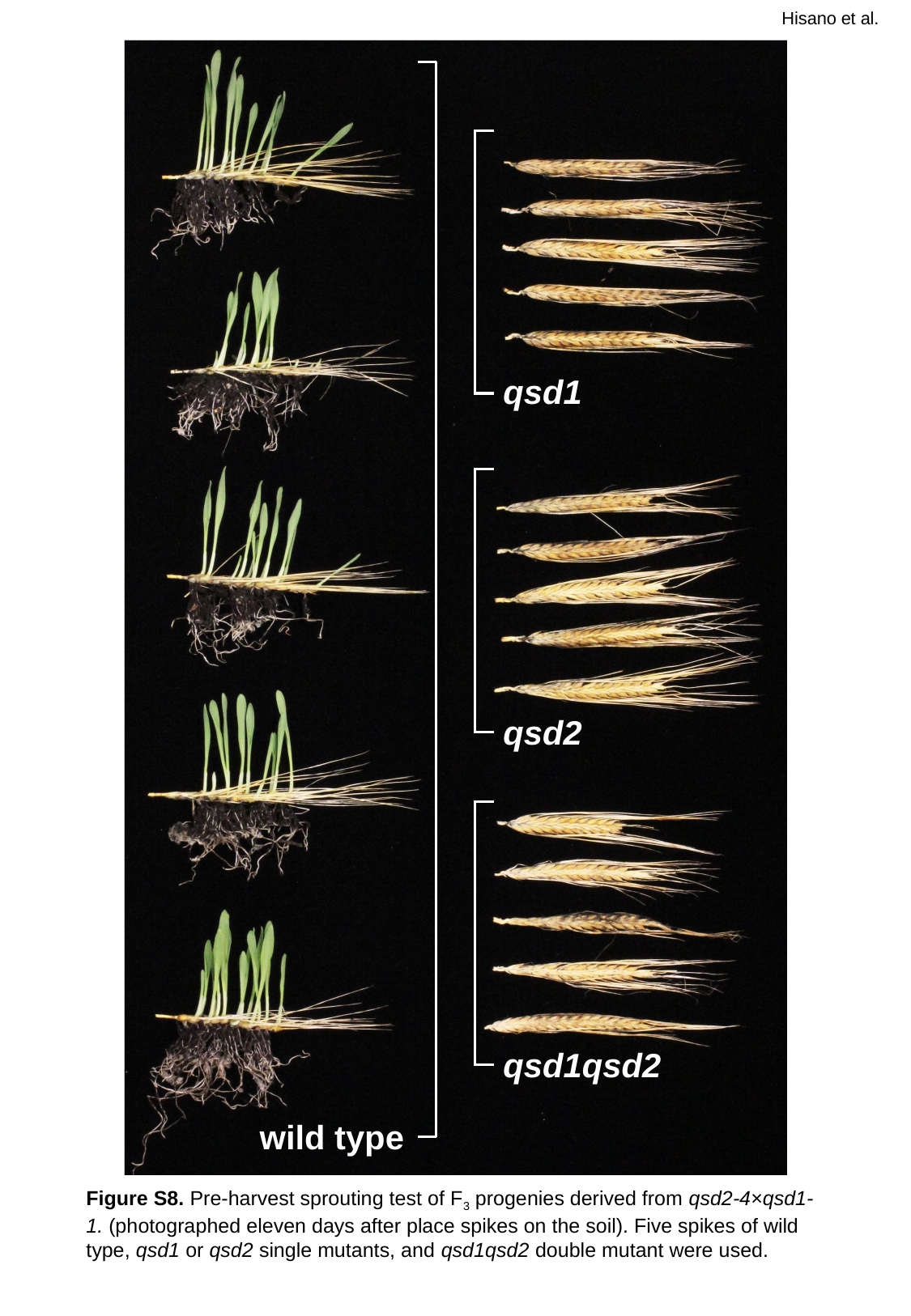

Hisano et al.
qsd1
qsd2
qsd1qsd2
wild type
Figure S8. Pre-harvest sprouting test of F3 progenies derived from qsd2-4×qsd1-1. (photographed eleven days after place spikes on the soil). Five spikes of wild type, qsd1 or qsd2 single mutants, and qsd1qsd2 double mutant were used.

## Slide 10
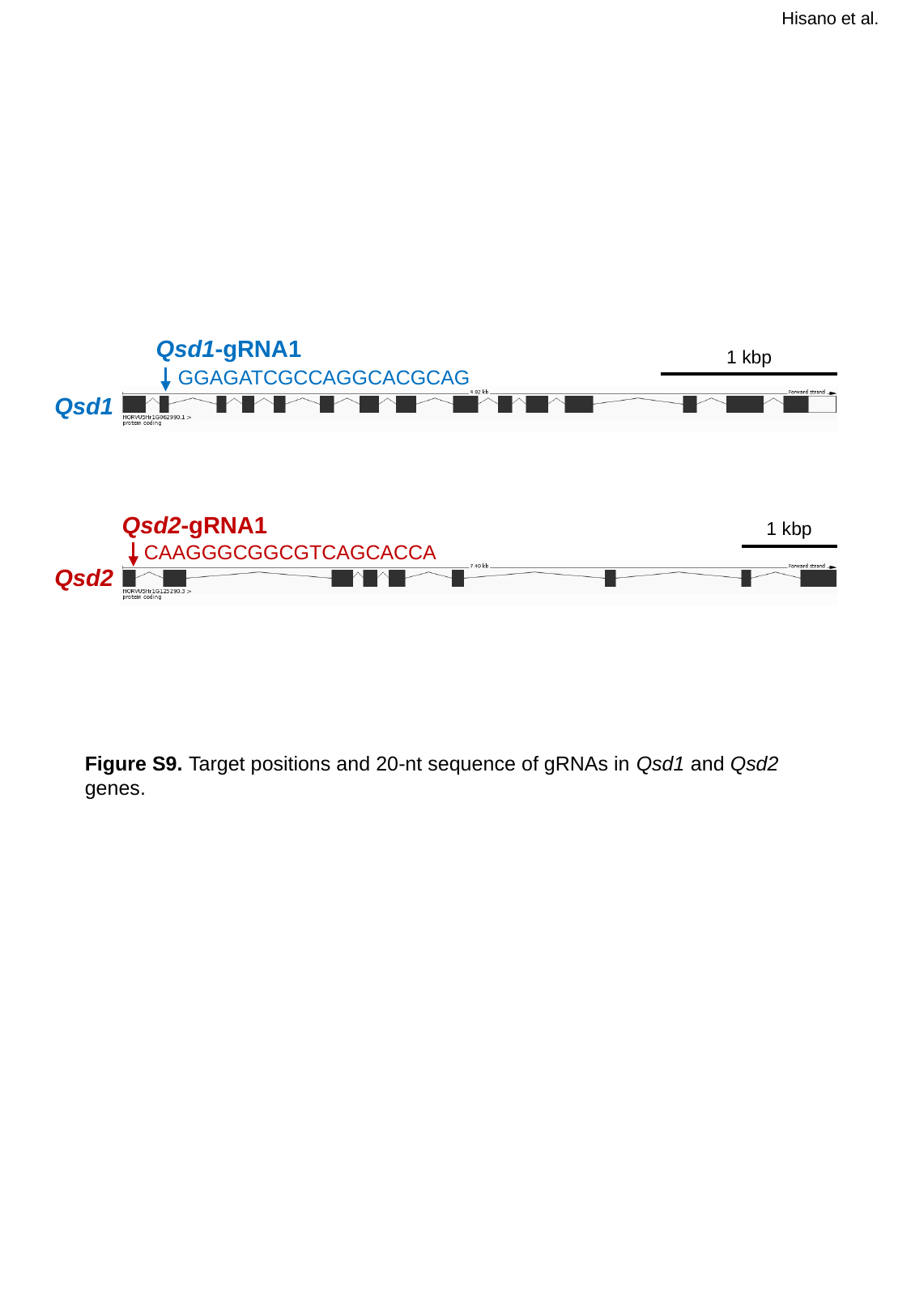

Hisano et al.
Qsd1-gRNA1
1 kbp
GGAGATCGCCAGGCACGCAG
Qsd1
Qsd2-gRNA1
1 kbp
CAAGGGCGGCGTCAGCACCA
Qsd2
Figure S9. Target positions and 20-nt sequence of gRNAs in Qsd1 and Qsd2 genes.

## Slide 11
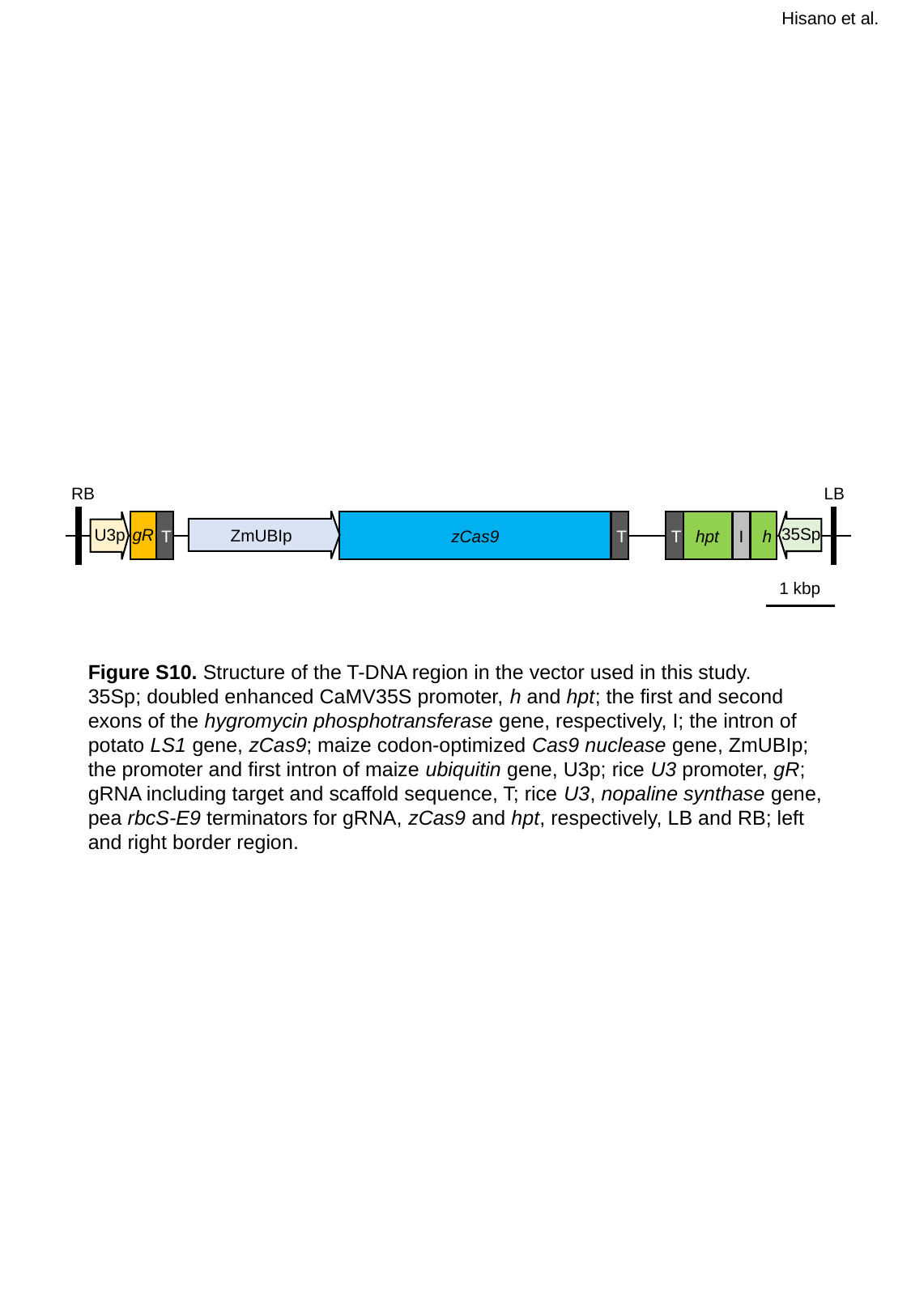

Hisano et al.
RB
LB
ZmUBIp
T
zCas9
T
T
hpt
I
h
35Sp
gR
U3p
1 kbp
Figure S10. Structure of the T-DNA region in the vector used in this study.
35Sp; doubled enhanced CaMV35S promoter, h and hpt; the first and second exons of the hygromycin phosphotransferase gene, respectively, I; the intron of potato LS1 gene, zCas9; maize codon-optimized Cas9 nuclease gene, ZmUBIp; the promoter and first intron of maize ubiquitin gene, U3p; rice U3 promoter, gR; gRNA including target and scaffold sequence, T; rice U3, nopaline synthase gene, pea rbcS-E9 terminators for gRNA, zCas9 and hpt, respectively, LB and RB; left and right border region.
